# Online exhaled propofol monitoring in normal‐weight and obese surgical patients

**DOI:** 10.1111/aas.14043

**Published:** 2022-02-19

**Authors:** Martin R. Braathen, Ivan Rimstad, Terje Dybvik, Ståle Nygård, Johan Ræder

**Affiliations:** ^1^ 56633 Institute of Clinical Medicine Faculty of Medicine University of Oslo Oslo Norway; ^2^ Department of Anaesthesiology Division of Critical Care Oslo University Hospital Oslo Norway; ^3^ 56633 Department of Informatics Faculty of Mathematics and Natural Sciences University of Oslo Oslo Norway; ^4^ 56633 Department of Biostatistics Institute of Basic Medical Sciences Oslo Centre for Biostatistics and Epidemiology University of Oslo Oslo Norway

## Abstract

**Background:**

Ion mobility spectrometry (IMS) allows for online quantification of exhaled propofol concentrations. We aimed to validate a bedside online IMS device, the Edmon^®^, for predicting plasma concentrations of propofol in normal‐weight and obese patients.

**Methods:**

Patients with body mass index (BMI) >20 kg/m^2^ scheduled for laparoscopic cholecystectomy or bariatric surgery were recruited. Exhaled propofol concentrations (C_A_), arterial plasma propofol concentrations (C_P_) and bispectral index (BIS) values were collected during target‐controlled infusion (TCI) anaesthesia. Generalised estimation equation (GEE) was applied to all samples and stable‐phase samples at different delays for best fit between C_P_ and C_A_. BMI was evaluated as covariate. BIS and exhaled propofol correlations were also assessed with GEE.

**Results:**

A total of 29 patients (BMI 20.3–53.7) were included. A maximal *R* ^2^ of 0.58 was found during stable concentrations with 5 min delay of C_A_ to C_P_; the intercept *a* = −0.69 (95% CI −1.7, 0.3) and slope *b* = 0.87 (95% CI 0.7, 1.1). BMI was found to be a non‐significant covariate. The median absolute performance error predicting plasma propofol concentrations was 13.4%. At a C_A_ of 5 ppb, the model predicts a C_P_ of 3.6 μg/ml (95% CI ±1.4). There was a maximal negative correlation of *R* ^2^ = 0.44 at 2‐min delay from C_A_ to BIS.

**Conclusions:**

Online monitoring of exhaled propofol concentrations is clinically feasible in normal‐weight and obese patients. With a 5‐min delay, our model outperforms the Marsh plasma TCI model in a post hoc analysis. Modest correlation with plasma concentrations makes the clinical usefulness questionable.

**Editorial Comment**

Measurement of exhaled propofol concentration and correlation to drug pharmacodynamics for patients in different weight categories is needed to see if the tool has general clinical value. This trial provides further experience with this method.

## 1 INTRODUCTION

There is a considerable variability in the relationship between a given iv dose of an anaesthetic drug and the effect. This is partly due to the variability in drug plasma concentration, which determines the concentration acting on individual cells in the central nervous system for effect. The present target control infusion (TCI) devices use pharmacokinetic (PK) models based on plasma measurements in the study populations, extrapolated for dosing in individual patients. Even the best TCI models will have performance errors due to inter‐individual variability.^1^ Morbid obesity poses an additional challenge due to non‐linear increases in both volume of distribution and drug clearance.^2^, ^3^ Truly individualised dosing would be better aided by some sort of online concentration monitoring. Bayesian optimisation of TCI based on individual bedside plasma concentration analysis may improve model bias, though inconvenient in everyday clinical practice.^4^

For inhalational anaesthetic agents, for decades we have been able to monitor alveolar gas concentrations in volume percent, as a reflection of partial pressure and concentrations in plasma. Such online concentration monitoring has not been available for dosing of intravenous drugs in routine clinical care.^5^ A basic amine with a p*K*_a_ of 11.1, propofol undergoes significant first‐pass uptake in the lungs; 28% in human patients.^6^, ^7^ Nearly all of that propofol eventually returns unchanged to the circulation except for trace amounts eliminated in expired breath.^8^ With a boiling point of 256℃ and a vapour pressure of 0.01 mmHg at 25℃, propofol has low volatility and gas concentrations are present in parts per billion (ppb).^9^ However, several groups have demonstrated that exhaled propofol may be identified, quantified and correlated to plasma concentrations using a variety of technologies.^10^, ^11^, ^12^, ^13^ Of these, Ion mobility spectrometry (IMS) may be the most promising method for routine use.^14^, ^15^ Refinement of the IMS technology combined with a multiple capillary column (MCC‐IMS) for handling humid gas has led to the launch of a CE‐marked, bedside, online drug monitor for quantifying propofol concentrations in exhaled air at a rate of once per minute, the Edmon^®^ (B. Braun Melsungen AG, Germany).^16^, ^17^, ^18^ Obesity alters respiratory physiology, including reduced functional residual capacity with risk of atelectases and ventilation‐perfusion mismatch.^2^ Thus, the suitability of MCC‐IMS technology in obese patients is unclear.

Our primary aim was to determine whether this device more accurately predicts plasma concentrations of propofol in normal‐weight and obese surgical patients, compared with a TCI model. Second, we investigated the correlation with bispectral index (BIS) to evaluate the pharmacodynamic effect in relation to exhaled propofol.

## 2 MATERIALS AND METHODS

### 2.1 Approvals and inclusion criteria

The study was registered with Clinical Trials (clinicaltrials.gov, identifier: NCT03817541), and approval was granted by the South‐East Norway regional ethics committee (helseforskning.etikkom.no, ref: 2017/2401). Written informed consent was obtained from adult patients scheduled for elective laparoscopic surgery, either cholecystectomy or bariatric surgery; the latter including either gastric bypass, mini gastric bypass or gastric sleeve. Patients of both sexes, aged 18–60 years and body mass index (BMI) >20 kg/m^2^ were eligible for inclusion.

### 2.2 Induction and maintenance of general anaesthesia

Anaesthesia was administered per standard operating procedure at our institution and all patients received premedication with 10 mg oxycodone po and 10 mg metoclopramide po in the morning of the day of surgery. Before induction, they were given glycopyrronium bromide 0.2 mg iv and esomeprazole 40 mg iv.

For patients with a BMI >30 kg/m^2^, propofol was dosed by adjusted body weight (ABW). ABW was determined by ideal body weight (IBW, kg) calculation from: height in cm—100 for men and height in cm—105 for women, adding 40% of the difference from total body weight (TBW). Thus, ABW = IBW + 0.4(TBW − IBW).^19^ Remifentanil was dosed by total body weight (TBW), maximum 120 kg; rocuronium was dosed to ABW.

Anaesthesia was induced and maintained with propofol by plasma‐controlled target control infusion (TCI), using the Marsh protocol with plasma target initially set at 6 μg/ml, then after endotracheal intubation at 3 μg/ml.^20^ For nociceptive input control, remifentanil effect‐site TCI (Minto) was started simultaneously with propofol, with an initial plasma target of 5 ng/ml, later adjusted to surgical needs at the discretion of the attending anaesthesiologist.^21^ Rocuronium 0.6 mg/kg was given to facilitate intubation (Shiley Hi‐Contour Oral/Nasal Tracheal Tube Cuffed). The patients were ventilated on a low‐flow (1 L/min) ventilator circuit with a tidal volume of 6 ml/kg ideal body‐weight, frequency 14/min and PEEP 5 cm H_2_O.

The patients received dexamethasone 16 mg iv after induction. Twenty minutes before the end of surgery, they were given paracetamol 1 g iv, ondansetron 4 mg iv, droperidol 1.25 mg iv and ketorolac 30 mg iv. By the end of the case, neuromuscular block was reversed by glycopyrrolate 0.4 mg + neostigmine 2.5 mg iv, and patients received fentanyl dosed at the discretion of the attending anaesthesiologist, typically 100 μg iv.

The patients were monitored with five‐lead ECG, pulsoxymetry, capnography, bispectral index monitoring (BIS) and train‐of‐four (TOF) measurements. Immediately after induction, a radial artery line was placed for blood sampling and invasive blood pressure recordings.

### 2.3 Surgical conditions

All patients were operated laparoscopically, in lithotomy reverse Trendelenburg position. Intra‐abdominal pressures were kept at 12 mmHg, increased to a maximum of 16 mmHg, at the discretion of the surgeon.

### 2.4 Exhaled propofol monitoring

**FIGURE 1:**
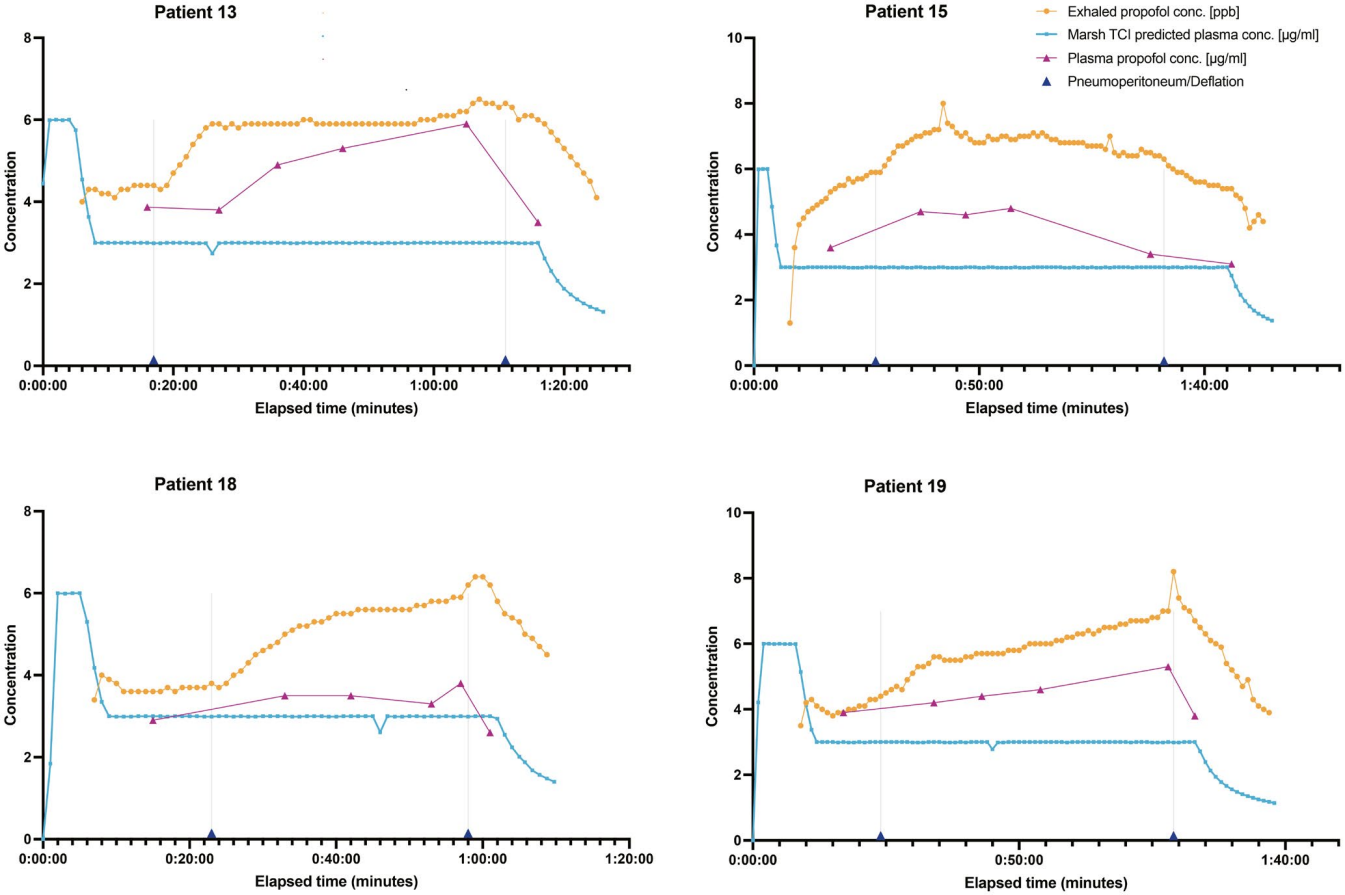
Exhaled propofol concentrations, plasma propofol concentrations, Marsh plasma target TCI predicted concentrations and time points of peritoneal inflation and deflation from four study patients

An illustration of the experimental setup is given as a supplementary online content; Digital Figure 1. The Edmon^®^ is a multiple capillary column ion mobility spectrometry device (MCC‐IMS). IMS technology allows identification and quantification of ionised compounds based on their mobility in an inert drift gas chamber. Passing the gas sample through a heated MCC rapidly isolates humidity and pre‐separates analytes in a complex gas, such as exhaled air, shortening measurement time and mitigating errors of analysis caused by humidity.^22^, ^23^ The Edmon^®^ was set up with synthetic air as carrier and drift gas, with a purity of ≥99.999% (Synthetic air HiQ 5.0; AGA). The gas was passed through an external filter (Air Liquide). New ventilation tubing (CareFusion Vital Signs, BD) was mounted prior to each study patient. Microbial filters (Iso‐Gard HepA Light, Teleflex) were placed at the inspiratory and expiratory ports of the anaesthesia machine only, as directed by the IMS device manufacturer. Side stream breath samples were drawn from the endotracheal tube via a T‐piece to the IMS device through a 1.8‐m long tube made of tetrafluoroethylene‐perfluoro copolymer (PFA) (Bohlender GmbH). The T‐piece was made of polysulfone (PSU) (VBM Medizintechnik GmbH). The IMS device draws air from the ventilation circuit for 20 s and spends 40 s on analysis. A new propofol concentration value is thus calculated and displayed, numerically and graphically in parts per billion (ppb), once every minute.

Exhaled propofol concentration (C_A_) values, blood sample time points, BIS values and TCI infusion data were harvested with dedicated software running on a medical grade PC (CLINEDMON, B. Braun Melsungen AG, Germany).

### 2.5 Propofol plasma samples

Four or six arterial blood samples were collected from each patient during anaesthesia. The first sample was taken immediately after intubation—before reducing the propofol plasma target from 6 to 3 μg/ml. The second sample was taken 10 min after intubation, followed by 1–3 samples during pneumoperitoneum, taken at approximately 10 min intervals. A final sample was taken after abdominal deflation—before stopping the propofol infusion.

Blood samples were immediately placed on ice and plasma separated within 60 min of sampling. Plasma samples were stored at −15–82°C until analysis, which was completed within 3 months of sampling. Plasma propofol concentrations (C_P_) were quantified by automated solid‐phase extraction liquid chromatography coupled mass spectrometry.^24^

### 2.6 Statistical analysis

**FIGURE 2:**
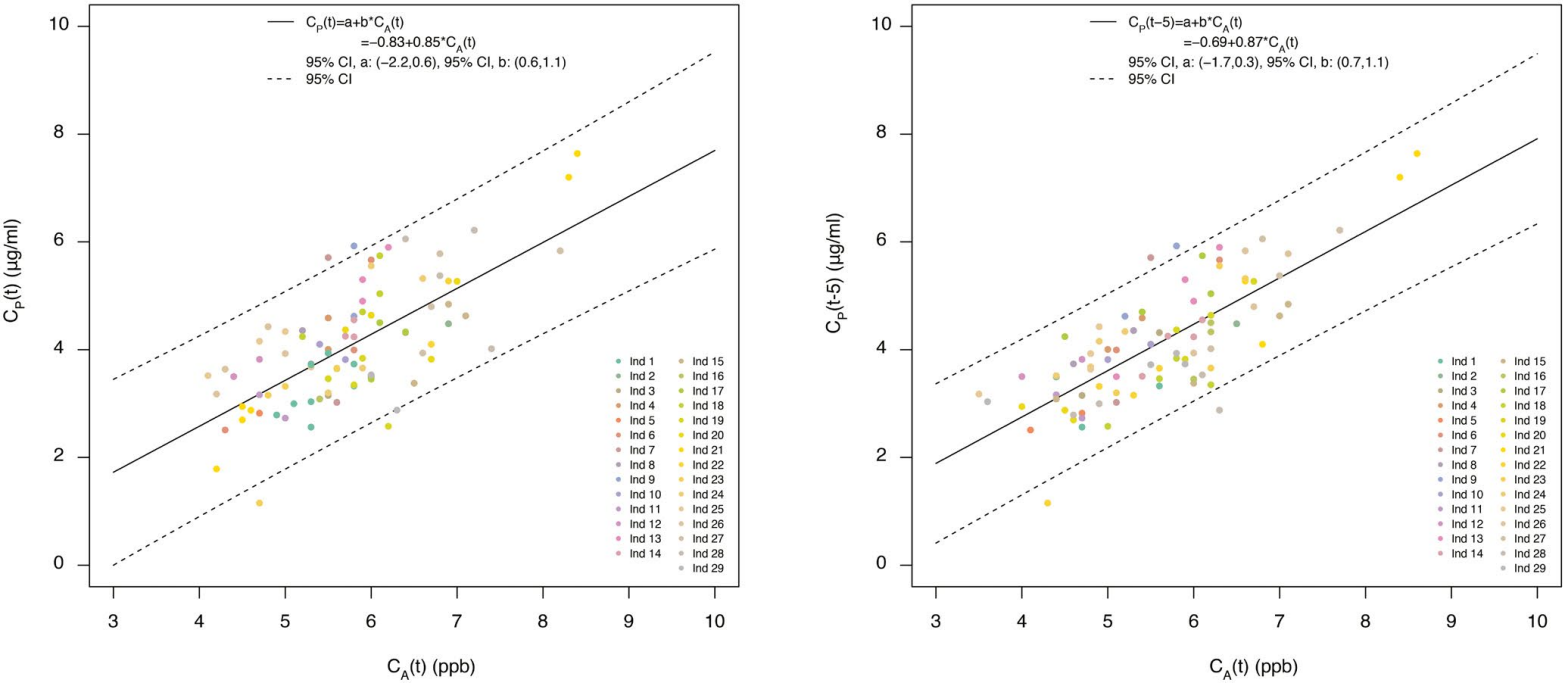
(A) Generalized estimating equation (GEE) with propofol concentration in exhaled air (C_Α_) as dependent variable and propofol concentration in plasma (C_Ρ_) as dependent variable. The first two plasma samples from each patient have been omitted. (B) Final GEE model of propofol concentrations in exhaled air and plasma, with first two plasma samples omitted and five minutes delay of (C_Α_) to (C_Ρ_)

Plotting of concentration curves was done in GraphPad Prism version 9.1.0 for MacOS, GraphPad Software, San Diego, California, USA, ‘www.graphpad.com’. Statistics were done in R, version 4.0.2.^25^ The longitudinal propofol measurements were modelled by generalised estimating equation (GEE) using the geeglm function in the geepack R library.^26^, ^27^ To determine the most appropriate correlation structure, models were calculated with all structures available in the geeglm function (i.e. ‘independence’, ‘exchangeable’, ‘ar1’, ‘unstructured’). The ‘exchangeable’ variance structure was thus chosen according to the quasi‐likelihood information criterion using the QIC function in the geepack library. In the GEE model, we assumed a linear relationship between the predictor variable and the response. The linearity assumption was examined by plotting Pearson residuals against fitted values, and the assumption was not invalidated (Digital Figure 2).

To determine delays of propofol concentrations in exhaled air (C_A_) to concentrations in plasma (C_P_), GEE was done with C_A_ as independent variable and C_P_ as dependent variable using delays of C_A_ to C_P_ from 0 to 10 min. Three data sets were used:

- All plasma samples included
- First plasma sample omitted
- Two first plasma samples omitted

Analyses with omitted samples done since the early phase presumably have less stable concentrations with non‐equilibrium between plasma and exhaled air when compared with samples taken after prolonged infusion at a steady plasma target level. The model with largest *R* ^2^ was selected, using the marginal *R* ^2^ formulated for GEE models by Zheng.^28^ To test if the relationship between C_A_ and C_P_ was dependent on body size, BMI was included as a covariate in the GEE model.

Performance error (PE) for each plasma sample and corresponding exhaled concentration as well as predicted plasma concentration by the Marsh TCI model used in the study wereassessed as follows:

$$\text{PE}=\left(C_{P}‐C_{\text{pred}}\right)/C_{\text{pred}}\times 100\%,$$

where C_P_ is the measured plasma concentration of propofol and C_pred_ is the concentration predicted by our models based on the Edmon measurements or the TCI model. The median performance error (MdPE) and median absolute performance error (MdAPE) were used to evaluate predictive bias and inaccuracy of both the Edmon and the Marsh TCI model as they were employed in the study.

In order to determine delays of BIS values to C_A_, GEE was done using the same correlation structure as above on the whole data set, with C_A_ as independent variable and BIS as dependent variable, using delays of BIS to C_A_ from 0 to 10 min.

## 3 RESULTS

### 3.1 Data set

The data set was collected from 29 patients with a total of 150 plasma propofol samples and 2646 exhaled propofol concentration observations. Five patients were of normal weight (BMI 18.5–24.9), eight were classified as overweight (BMI 25–29.9) and 16 as obese (BMI >30) (Table 1).^29^

**TABLE 1:** Patients' characteristics. Values are mean, SD (range)

	(*n* = 29)
Age (years)	38.9 SD 10.7 (18–56)
Sex (Male/female) (*n*)	6/23
Weight (kg)	102 SD 34.6 (54–185.8)
Height (cm)	167 SD 8.9 (158–192)
BMI (kg/m^2^)	36.2 SD 10.8 (20.3–53.7)
Duration of surgery (minutes)	66 SD 26 (41–134)
Total dose of propofol (mg)	966 SD 231 (714–1764)
Total dose of remifentanil (μg)	1248 SD 342 (727–2100)

**FIGURE 3:**
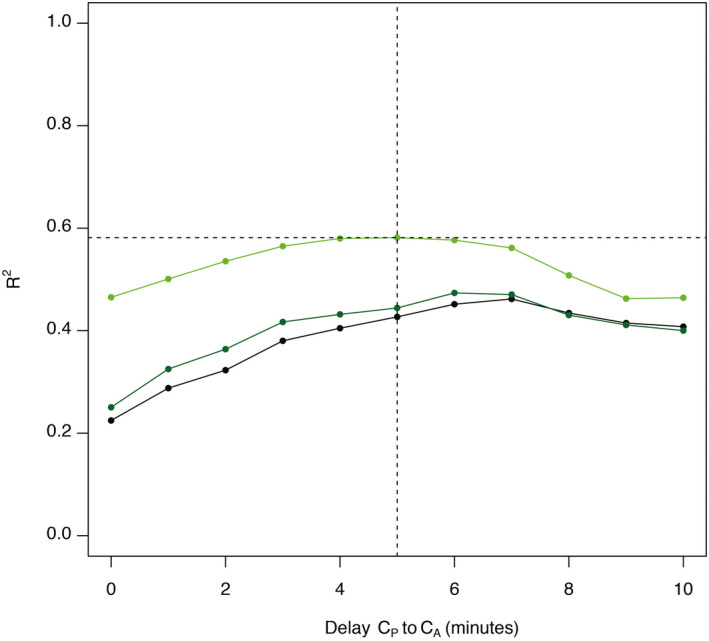
Coefficient of determination (*R* ^2^) of exhaled and plasma propofol concentrations at different delays. GEE with all samples included is marked in black, with the first plasma sample omitted in dark green and with the first two plasma samples omitted in bright green

Plots of exhaled propofol and plasma propofol concentrations from four typical patients are shown in Figure 1. Plots from all 29 patients are available as supplementary online content (Digital Figure 3).

### 3.2 Propofol concentrations in exhaled air and plasma

Linear regression with GEE on the entire set of plasma concentrations vs exhaled concentrations yielded poor correlation, with *R* ^2^ = 0.23. When calculations were repeated omitting the first plasma sample, the result was 0.25. Excluding the first two plasma samples, *R* ^2^ was 0.47 (Figure 2A). Linear regression with increasing delays of C_A_ to C_P_ yielded a maximal coefficient of determination with 5‐min delay (Figure 3). With a delay of 5 min and omitting the first two plasma samples, *R* ^2^ increased to 0.58, with intercept *a* = −0.69 (95% CI −1.7, 0.3) and slope *b* = 0.87 (95% CI 0.7, 1.1) (Figure 2B). For example, given these conditions, a C_A_ of 5 ppb predicts a C_P_ of 3.6 μg/ml with a 95% confidence interval of ±1.4, i.e., between 2.2 and 5.0 μg/ml. BMI was found to be a non‐significant covariate (*p* = 0.051) and was not included in the final model.

MdPE and MdAPE were lower for both GEE models than for the Marsh plasma‐controlled TCI model (Table 2).

**TABLE 2:** Predictive Median Performance Error (MdPE) and Median Absolute Performance Error (MdAPE) of Marsh plasma‐controlled TCI and two prediction models based on exhaled air concentration monitoring. Values are median (25th, 75th percentile). The first two plasma samples from each patient were omitted before evaluation

	MdPE (%)	MdAPE (%)
Plasma‐controlled Marsh TCI	−24.9 (−37.2, −13.6)	25.6 (15.4, 38.3)
Exhaled air, no delay	−8.0 (−23.3, 9.9)	17.3 (8.5, 26.9)
Exhaled air, 5 min delay	0.4 (−10.9, 16.5)	13.4 (7.0 ,19.6)

### 3.3 C_A_:BIS

BIS values were recorded from 25 patients. (Digital Figure 4) A negative correlation between C_A_ and BIS was found, with *R* ^2^ = 0.42. Applying delays improved the correlation only modestly. A 2‐min delay produced the best correlation, with *R* ^2^ = −0.44 (Digital Figure 5).

## 4 DISCUSSION

The exhaled drug monitor Edmon^®^ predicted plasma concentrations of propofol with greater precision and accuracy than the Marsh TCI model during stable propofol plasma concentrations when a delay of C_A_ to C_P_ of 5 min was applied. We found moderate negative correlation of C_A_ to BIS values.

Initial calculations on the whole data set produced low *R* ^2^. When we assumed plasma concentrations to be unstable after adjustments of propofol TCI targets and did model calculations on samples taken at approximately 20 min after the plasma TCI target was stabilised the correlations improved. A delay of 5 min between plasma and exhaled concentrations further increased *R* ^2^. We hypothesised that body size could be a covariate in the model, due to alterations in ventilatory mechanics in obese subjects. BMI was found to be a non‐significant covariate.

We conducted the study in a regular operating room setting on scheduled patients of a wide range of body sizes. The surgical categories, cholecystectomy and bariatric surgery were chosen for consistent perioperative conditions in all patients, including body positioning, laparoscopic technique, peritoneal insufflation pressures, mode of anaesthesia and duration of surgery. In the individual patient plots, both C_A_ and C_P_ are seen to decrease after the point of peritoneal deflation, despite the TCI settings being maintained unchanged until the last blood sample was taken. These concentration decreases following abdominal deflation may be secondary to increased cardiac output following deflation.^30^ Laparoscopic insufflation of CO_2_ at 12 mmHg has been shown to increase hepatic blood flow.^31^ In our study, insufflation pressures up to 16 mmHg were used and may have influenced hepatic clearance of propofol.

Adhesion of propofol to materials in contact with the exhaled air before it reaches the Edmon^®^ may potentially result in underestimation of exhaled values in the early phase of a propofol‐based procedure and overestimation when reducing or ending the infusion. Polyurethane or tubing made of silicone may absorb most of the propofol molecules and are thus unsuitable.^32^ Few authors describe either the types of materials in the sampling tubes or the airway devices used. We set up the breathing circuit with previously tested materials only, as directed by the manufacturer. The Edmon device is so far not approved for use with laryngeal mask airways.

The modest *R* ^2^ of 0.58, even when including only assumed stable‐phase C_P_ and applying a 5‐min delay, suggests limitations with the method as used in our study.

Several groups have specified sampling expired air or end‐tidal air.^9^, ^33^ This may explain why two studies with a statistical approach similar to ours produced higher *R* ^2^; Hornuss et al. synchronised their ion molecule reaction mass spectrometer with a separate capnograph for approximate end‐expiratory sampling while Perl et al did CO_2_‐controlled sampling with their MCC‐IMS device.^14^, ^34^ Colin et al. recorded the median expiratory breath signal during 30 s intervals.^13^ The Edmon^®^ draws a mixed‐breath side‐stream sample continuously over 20 s. In our low‐flow circuit system with 10–20 min allowed for establishment of a steady‐state situation, we may assume the inspiratory to expiratory fluctuations in propofol concentrations to be minimal. This may differ with high ventilator gas‐flow and during induction, dose‐adjustments or at the end of anaesthesia. Further, we may not have achieved as steady concentrations with the Marsh TCI model in our diverse patient group as in the aforementioned studies.

For exhaled drug monitoring to be useful, the clinician may want to know three things: (1) the corresponding concentration in plasma; (2) the delay in equilibration of concentrations in plasma with alveolar air and (3) the correlation and delay with pharmacodynamic variables i.e., effect‐site concentrations.

Ziaian et al. and Colin et al. have presented conversion factors from C_P_ to C_A_ of 2.71 ppb/ml/μg and 3.56 ppb/ml/μg.^13^, ^35^ Kreuer et al. calculated a much lower conversion factor of 0.66 ppb/ml/μg, which may be disregarded since they used a calibration procedure not presently recommended.^18^, ^36^ Chen et al. demonstrated interindividual variability in the blood/exhaled partial pressure ratio (R_BE_) of propofol and have proposed a method of individualised calculation of this ratio. However, this takes up to 50 min to determine per patient using a gas chromatograph–surface acoustic wave sensor.^12^ A practical interpretation of exhaled concentration data during clinical use of the Edmon^®^ during pseudo‐steady‐state plasma concentrations is approximately 1.4 ppb/ml/μg, as derived from our model.

A potential objection to our study is that we did not calculate a first‐order rate constant for the delay of C_A_ to C_P_ by pharmacokinetic modelling. In our view, this is not a prerequisite for practical application of exhaled drug monitoring. An early study by Takita et al. using proton transfer mass spectrometry reported a delay of 333 s to maximum propofol concentrations in expired breath after a bolus injection, apparently determined by visual approximation of the data.^11^ Kreuer et al., Ziaian et al. and Colin et al. have all produced time constants for the delay in equilibration of concentration between plasma and breath by compartmental PK model approaches. Kreuer et al. used the same IMS technology as ours, while Ziaian et al. utilised an electrochemical sensor and Colin et al. an ion molecule reaction mass spectrometry device. The delay constants (k_e_0_lung_) produced were 0.209, 0.155 and 0.152, respectively. Colin et al. aimed to use exhaled propofol measurements to increase dosing accuracy by Bayesian adaptation of the Eleveld TCI model. We believe a more practical approach is preferable wherein the online exhaled drug monitor assists the clinician in much the same way as end‐tidal concentration monitoring during volatile gas anaesthesia. During maintenance of TCI‐based anaesthesia, when plasma concentrations are near steady‐state, the exhaled drug monitor may be used as supplement to improve on the predictions of the TCI model. The Edmon^®^ has a graphical display of concentration trend over time that will be useful for clinicians interpreting the currently displayed C_A_. However, a prospective validation of the GEE model we produced in a new patient population is needed before clinical implementation. Notably, the favourable bias and inaccuracy we present were produced on the same data originating the model, thus providing only an indication of the true performance of the Edmon.

We found a moderate negative correlation of plasma propofol (C_A_) to BIS values in our study during ongoing surgery and minor improvement from introducing the optimal delay of 2 min. In states of acute nociception or strong influence from opioids, the BIS may not be a reliable monitor of anaesthetic adequacy.^37^, ^38^ In such situations individual online monitoring of plasma propofol concentration, although incorporating a delay of 5 min as in our study, may be valuable for clinicians in ensuring an adequate depth of anaesthesia. We found moderate negative correlation of exhaled propofol and BIS for simultaneous measurements and minor improvement from introducing the optimal delay of 2 min. A limitation of our data set is the low resolution of 1 BIS value per minute and data missing from four patients. Hornuss et al. using ion molecule reaction mass spectrometry found maximum correlation between plasma and BIS after approximately 3.5 min.^39^

## 5 CONCLUSION

Online monitoring of exhaled propofol concentrations with a novel IMS device is feasible during stable plasma concentrations in adult normal‐weight and obese patients. Tested post hoc, our model predicts plasma concentration better than the Marsh plasma‐controlled TCI model. The modest correlation and 5‐min delay make the everyday clinical usefulness questionable.

## DECLARATION OF INTERESTS

MRB, IR, TD and JR received a travel grant from B. Braun Melsungen AG, Germany for training on the study device at Saarland University Hospital, Germany.

## AUTHOR CONTRIBUTIONS

MRB: Study design, data collection, writing up the first draft of the paper. IR: Study design, technical advisory, data collection. TD: Patient recruitment, data collection. SN: Statistical analysis, major paper revisions. JR: Study design, data analysis, major paper revisions.

## Supporting information

Supplementary Material

Supplementary Material

Supplementary Material

Supplementary Material

Supplementary Material

## References

[1] Vellinga R , Hannivoort LN , Introna M , et al. Prospective clinical validation of the Eleveld propofol pharmacokinetic‐pharmacodynamic model in general anaesthesia. Br J Anaesth. 2021;126:386‐394. 33317804 10.1016/j.bja.2020.10.027

[2] Schumann R . Anesthesia for the patient with obesity at https://www‐uptodate‐com.ezproxy.uio.no/contents/anesthesia‐for‐the‐patient‐with‐obesity?search=obesity%20anesthesia&source=search_result&selectedTitle=1~150&usage_type=default&display_rank=1 Accessed January 18, 2022

[3] Cortínez LI , la Fuente DN , Eleveld DJ , et al. Performance of propofol target‐controlled infusion models in the obese. Anesth Analg. 2014;119:302‐310. 24977639 10.1213/ANE.0000000000000317

[4] van den Berg JP , Eleveld DJ , De Smet T , et al. Influence of Bayesian optimization on the performance of propofol target‐controlled infusion. Br J Anaesth. 2017;119:918‐927. 29028925 10.1093/bja/aex243

[5] Hemmings HC , Egan TD . Pharmacology and Physiology for Anesthesia E‐Book. 2nd ed. Elsevier Health Sciences; 2018.

[6] He YL , Ueyama H , Tashiro C , Mashimo T , Yoshiya I . Pulmonary disposition of propofol in surgical patients. Anesthesiology. 2000;93:986‐991. 11020751 10.1097/00000542-200010000-00019

[7] Chen Y‐Z , Zhu S‐M , He H‐L , Xu J‐H , Huang S‐Q , Chen Q‐L . Do the lungs contribute to propofol elimination in patients during orthotopic liver transplantation without veno‐venous bypass? HBPD INT. 2006;5:511‐514. 17085334

[8] Müller‐Wirtz LM , Maurer F , Brausch T , et al. Exhaled propofol concentrations correlate with plasma and brain tissue concentrations in rats. Anesth Analg. 2020;132:110‐118. 10.1213/ANE.0000000000004701 32118620

[9] Grossherr M , Hengstenberg A , Meier T , Dibbelt L , Gerlach K , Gehring H . Discontinuous monitoring of propofol concentrations in expired alveolar gas and in arterial and venous plasma during artificial ventilation. Anesthesiology. 2006;104:786‐790. 16571975 10.1097/00000542-200604000-00024

[10] Harrison GR , Critchley ADJ , Mayhew CA , Thompson JM . Real‐time breath monitoring of propofol and its volatile metabolites during surgery using a novel mass spectrometric technique: a feasibility study. Br J Anaesth. 2003;91:797‐799. 14633747 10.1093/bja/aeg271

[11] Takita A , Masui K , Kazama T . On‐line monitoring of end‐tidal propofol concentration in anesthetized patients. Anesthesiology. 2007;106:659‐664. 17413902 10.1097/01.anes.0000264745.63275.59

[12] Chen X , Zhang XL , Liu L , et al. Gas chromatograph surface acoustic wave for quick real‐time assessment of blood/exhaled gas ratio of propofol in humans. Br J Anaesth. 2014;113:807‐814. 25012583 10.1093/bja/aeu193

[13] Colin P , Eleveld DJ , van den Berg JP , et al. Propofol breath monitoring as a potential tool to improve the prediction of intraoperative plasma concentrations. Clin Pharmacokinet 2015;55: 1‐13. 10.1007/s40262-015-0358-z 26715214

[14] Perl T , Carstens E , Hirn A , et al. Determination of serum propofol concentrations by breath analysis using ion mobility spectrometry. Br J Anaesth. 2009;103:822‐827. 19887534 10.1093/bja/aep312

[15] Liu Y , Gong Y , Wang C , et al. Online breath analysis of propofol during anesthesia: clinical application of membrane inlet‐ion mobility spectrometry. Acta Anaesth Scand. 2015;59:319‐328. 25565144 10.1111/aas.12448

[16] Kreuder AE , Buchinger H , Kreuer S , Volk T , Maddula S , Baumbach JI . Characterization of propofol in human breath of patients undergoing anesthesia. Int J Ion Mobil Spec. 2011;14:167‐175.

[17] Buchinger H , Kreuer S , Hellbrück R , et al. Minimal retarded Propofol signals in human breath using ion mobility spectrometry. Int J Ion Mobil Spec. 2013;16:185‐190.

[18] Maurer F , Walter L , Geiger M , et al. Calibration and validation of a MCC/IMS prototype for exhaled propofol online measurement. J Pharmaceut Biomed. 2017;145:293‐297. 10.1016/j.jpba.2017.06.052 28704718

[19] Servin F , Farinotti R , Haberer JP , Desmonts JM . Propofol infusion for maintenance of anesthesia in morbidly obese patients receiving nitrous oxide. A clinical and pharmacokinetic study. Anesthesiology. 1993;78:657‐665. 8466066 10.1097/00000542-199304000-00008

[20] Marsh B , White M , Morton N , Kenny GN . Pharmacokinetic model driven infusion of propofol in children. Br J Anaesth. 1991;67:41‐48. 1859758 10.1093/bja/67.1.41

[21] Minto CF , Schnider TW , Egan TD , et al. Influence of age and gender on the pharmacokinetics and pharmacodynamics of remifentanil. I. Model development. Anesthesiology. 1997;86:10‐23. 9009935 10.1097/00000542-199701000-00004

[22] Vautz W , Sielemann S , Baumbach JI . Determination of terpenes in humid ambient air using ultraviolet ion mobility spectrometry. Anal Chim Acta. 2004;513:393‐399.

[23] Vautz W , Baumbach JI . Exemplar application of multi‐capillary column ion mobility spectrometry for biological and medical purpose. Int J Ion Mobil Spec. 2008;11:35‐41.

[24] Maurer F , Shopova T , Wolf B , et al. Design and validation of an automated solid phase extraction liquid chromatography coupled mass spectrometry method for the quantification of propofol in plasma. J Pharmaceut Biomed. 2018;150:341‐346. 10.1016/j.jpba.2017.12.043 29287260

[25] R Core Team . R: A Language and Environment for Statistical Computing. 4th ed. 2020. https://www.R‐project.org

[26] Schober P , Vetter TR . Repeated measures designs and analysis of longitudinal data: If at first you do not succeed‐try, try again. Anesth Analg. 2018;127:569‐575. 29905618 10.1213/ANE.0000000000003511 PMC6072386

[27] Højsgaard S , Halekoh U , Yan J . The R package geepack for generalized estimating equations. J Stat Softw. 2005;15:1‐11.

[28] Zheng B . Summarizing the goodness of fit of generalized linear models for longitudinal data. Stat Med. 2000;19:1265‐1275. 10814976 10.1002/(sici)1097-0258(20000530)19:10<1265::aid-sim486>3.0.co;2-u

[29] Obesity: identification, assessment and management 2014 at https://www.nice.org.uk/guidance/cg189/chapter/1‐Recommendations Accessed April 20, 2021

[30] Atkinson TM , Giraud GD , Togioka BM , Jones DB , Cigarroa JE . Cardiovascular and ventilatory consequences of laparoscopic surgery. Circulation. 2017;135:700‐710. 28193800 10.1161/CIRCULATIONAHA.116.023262

[31] Meierhenrich R , Gauss A , Vandenesch P , Georgieff M , Poch B , Schütz W . The effects of intraabdominally insufflated carbon dioxide on hepatic blood flow during laparoscopic surgery assessed by transesophageal echocardiography. Anesth Analg. 2005;100:340‐347. 15673853 10.1213/01.ANE.0000143566.60213.0A

[32] Maurer F , Lorenz DJ , Pielsticker G , et al. Adherence of volatile propofol to various types of plastic tubing. J Breath Res. 2017;11:016009. 28049865 10.1088/1752-7163/aa567e

[33] Hornuss C , Dolch ME , Janitza S , et al. Determination of breath isoprene allows the identification of the expiratory fraction of the propofol breath signal during real‐time propofol breath monitoring. J Clin Monit Comput. 2013;27:509‐516. 23525901 10.1007/s10877-013-9452-7

[34] Hornuss C , Praun S , Villinger J , et al. Real‐time monitoring of propofol in expired air in humans undergoing total intravenous anesthesia. Anesthesiology. 2007;106:665‐674. 17413903 10.1097/01.anes.0000264746.01393.e0

[35] Ziaian D , Herrmann R , Kleiboemer K , et al. Pharmacokinetic modeling of the transition of propofol from blood plasma to breathing gas. 2014 IEEE International Symposium on Medical Measurements and Applications (MeMeA). IEEE; 2014:1‐5

[36] Kreuer S , Hauschild A , Fink T , Baumbach JI , Maddula S , Volk T . Two different approaches for pharmacokinetic modeling of exhaled drug concentrations. Sci Rep. 2014;4:661‐666. 10.1038/srep05423 PMC4067807 24957852

[37] Lysakowski C , Dumont L , Pellegrini M , Clergue F , Tassonyi E . Effects of fentanyl, alfentanil, remifentanil and sufentanil on loss of consciousness and bispectral index during propofol induction of anaesthesia. Br J Anaesth. 2001;86:523‐527. 11573626 10.1093/bja/86.4.523

[38] Wang LP , McLoughlin P , Paech MJ , Kurowski I , Brandon EL . Low and moderate remifentanil infusion rates do not alter target‐controlled infusion propofol concentrations necessary to maintain anesthesia as assessed by bispectral index monitoring. Anesth Analg. 2007;104:325‐331. 17242088 10.1213/01.ane.0000252966.03103.89

[39] Hornuss C , Wiepcke D , Praun S , Dolch ME , Apfel CC , Schelling G . Time course of expiratory propofol after bolus injection as measured by ion molecule reaction mass spectrometry. Anal Bioanal Chem. 2012;403:555‐561. 22370587 10.1007/s00216-012-5856-3

