# Supplementary figures and images for: Online exhaled propofol monitoring in normal‐weight and obese surgical patients
Source: Acta Anaesthesiol Scand. 2022 Feb 19;66(5):598–605. doi: 10.1111/aas.14043 (PMC9305953; doi:10.1111/aas.14043)

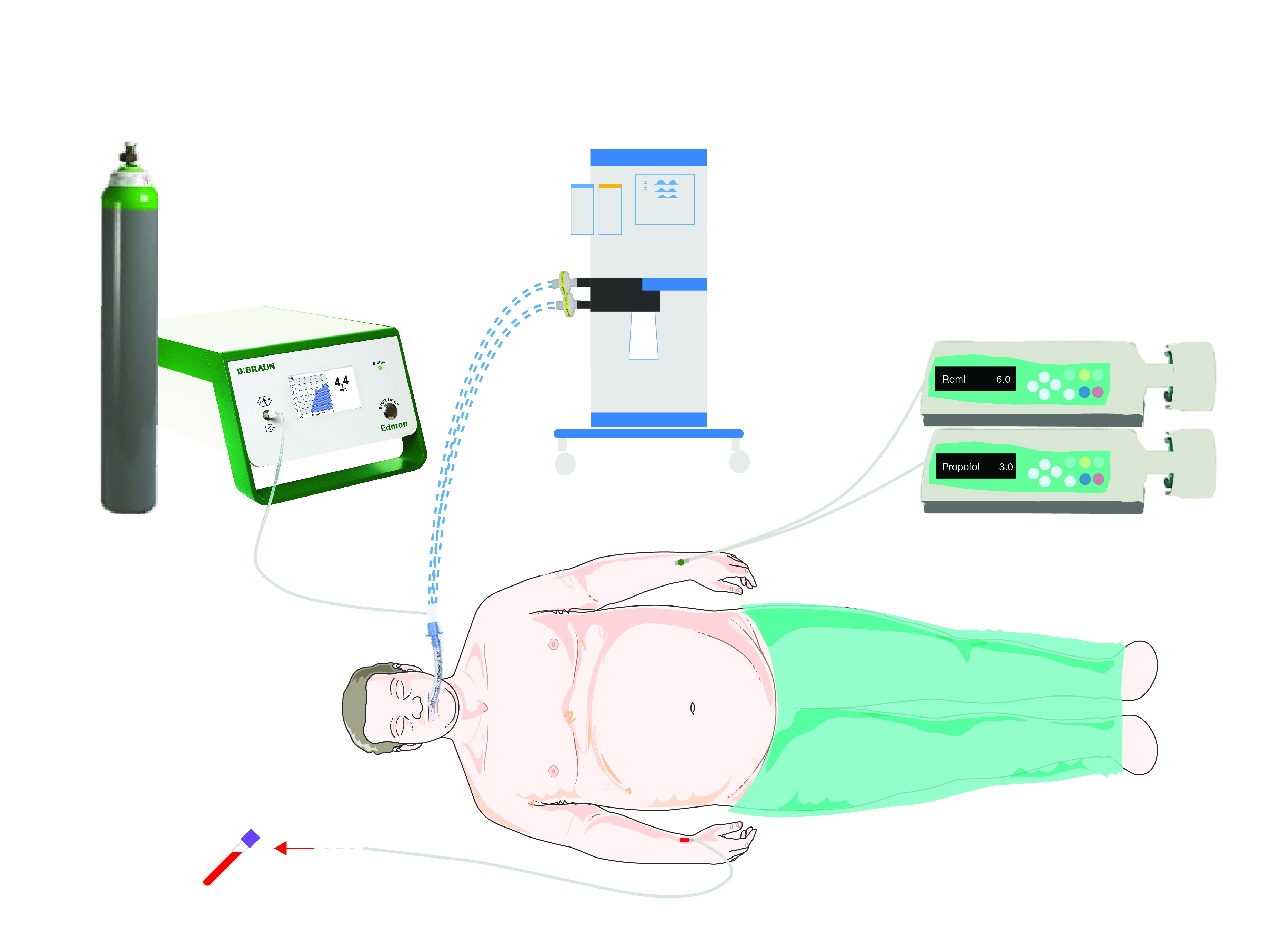

Supplement: Supplementary file 1 — Supplementary Material [file AAS-66-598-s002.jpg]

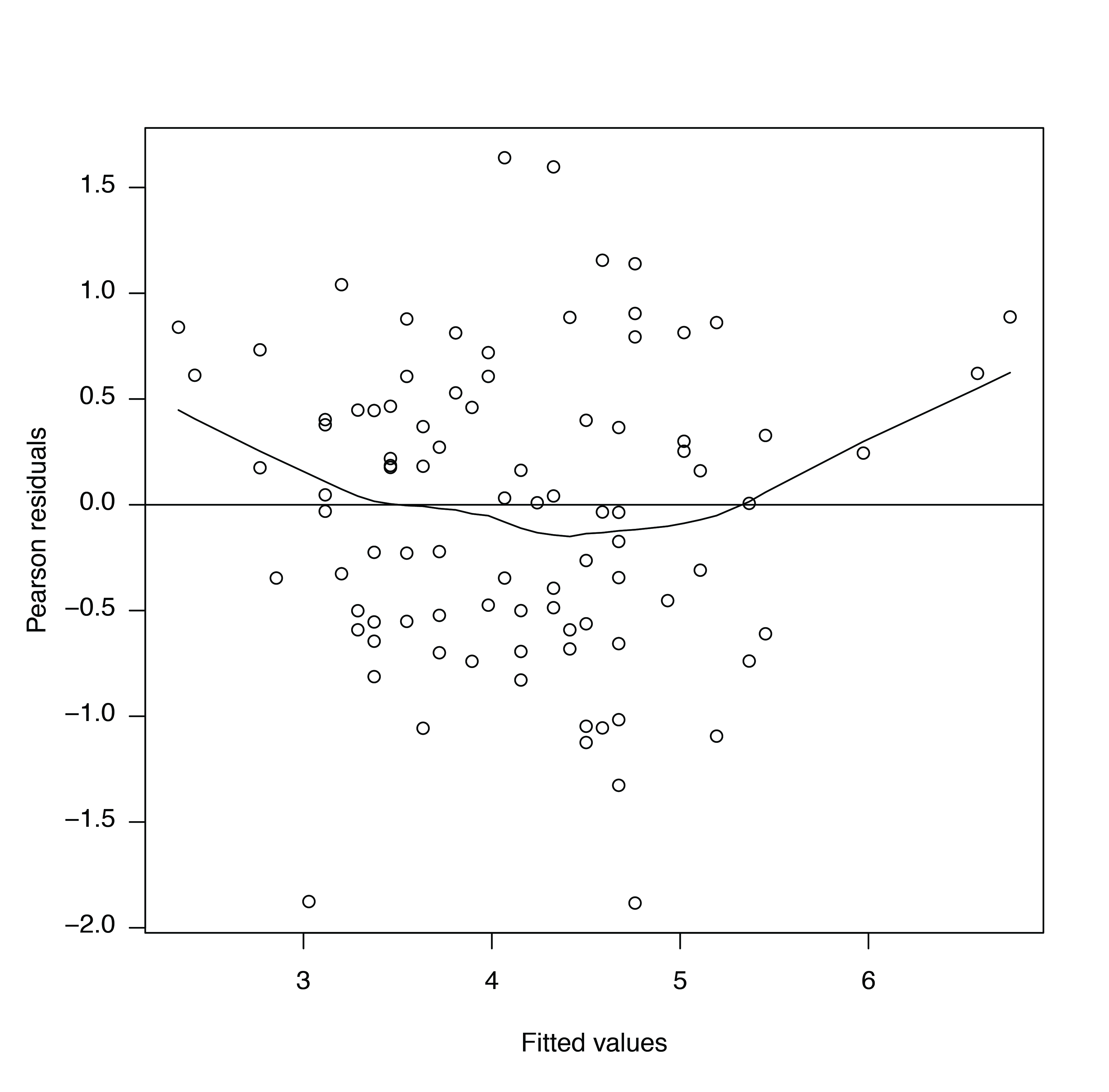

Supplement: Supplementary file 2 — Supplementary Material [file AAS-66-598-s001.jpg]

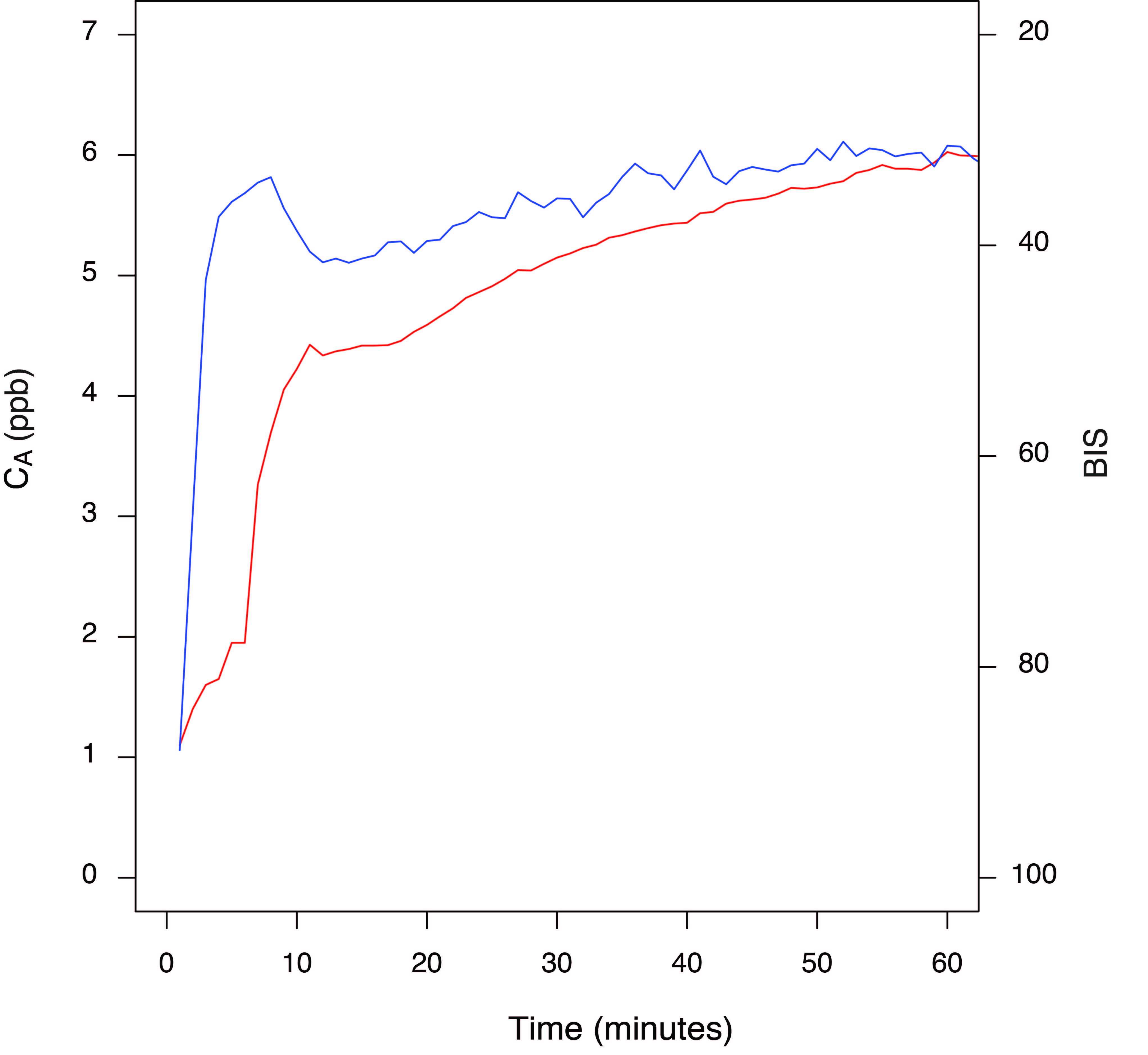

Supplement: Supplementary file 4 — Supplementary Material [file AAS-66-598-s003.jpg]

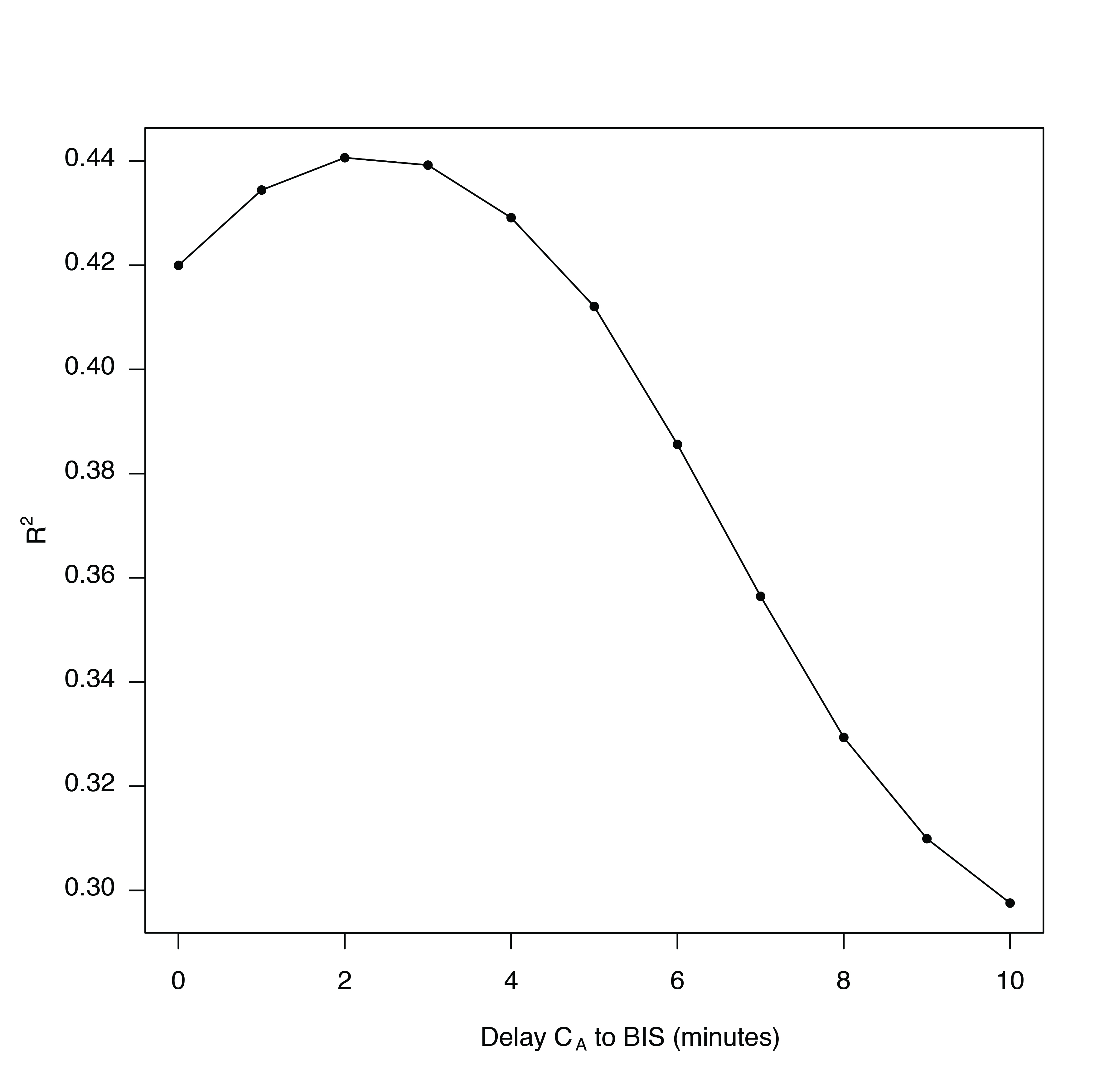

Supplement: Supplementary file 5 — Supplementary Material [file AAS-66-598-s005.jpg]
