# Supplementary material for: Online exhaled propofol monitoring in normal‐weight and obese surgical patients
Source: Acta Anaesthesiol Scand. 2022 Feb 19;66(5):598–605. doi: 10.1111/aas.14043 (PMC9305953; doi:10.1111/aas.14043)
Supplement: Supplementary file 3 — Supplementary Material [file AAS-66-598-s004.pdf]

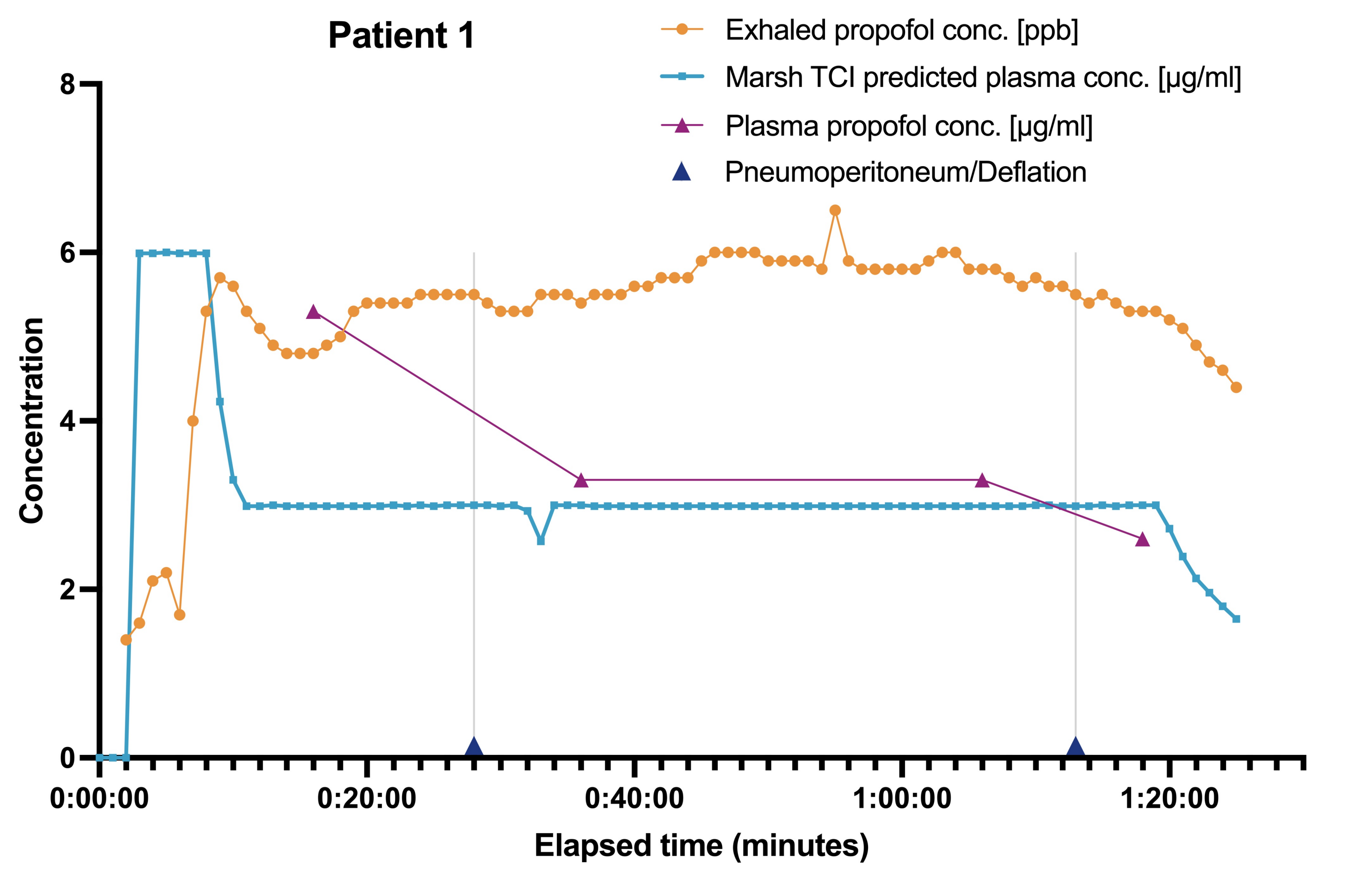

Patient 1
Exhaled propofol conc. [ppb]
Marsh TCI predicted plasma conc. [µg/ml]
Plasma propofol conc. [µg/ml]
Pneumoperitoneum/Deflation
Concentration
0
2
4
6
8
0:00:00
0:20:00
0:40:00
1:00:00
1:20:00
Elapsed time (minutes)

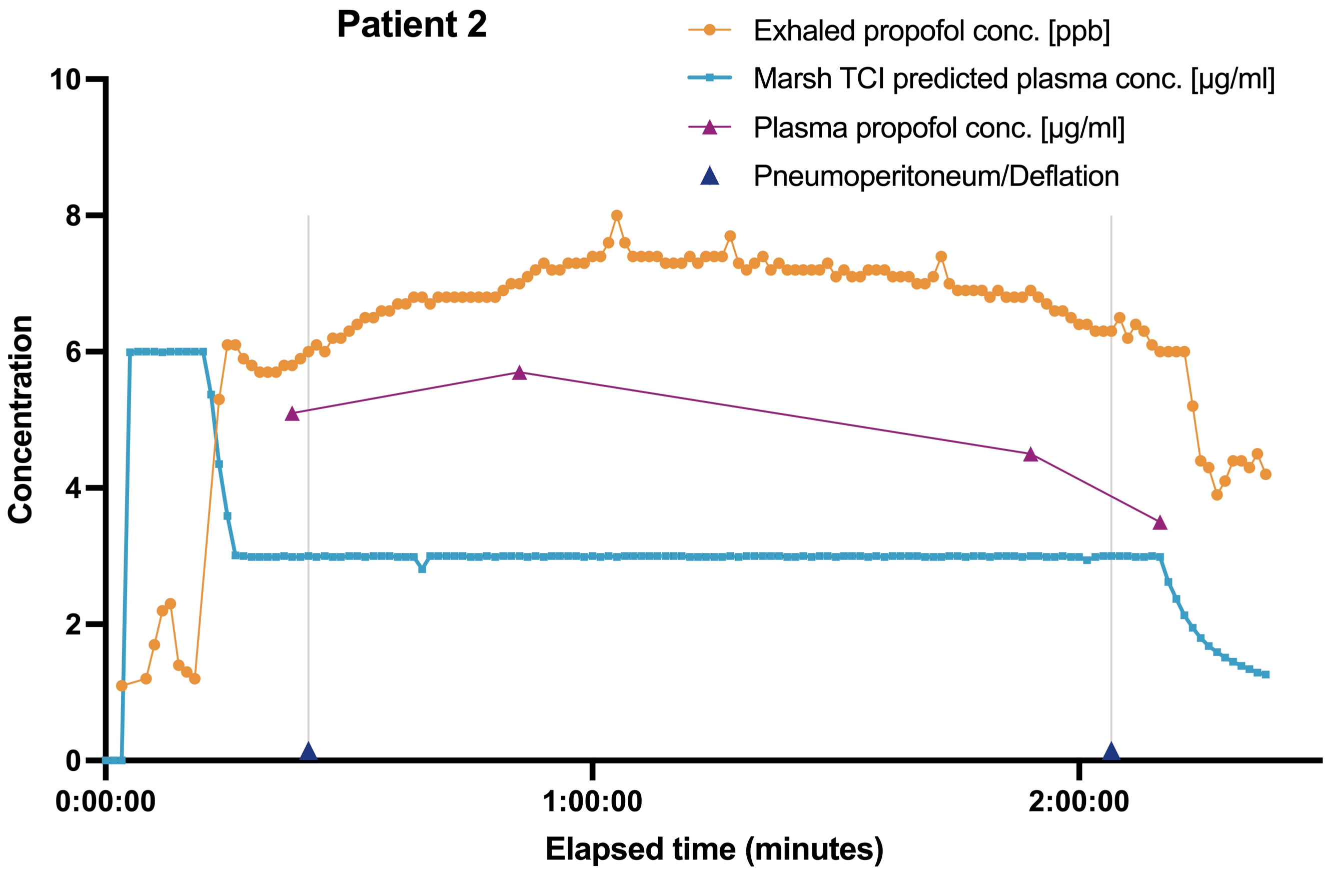
Patient 2
Exhaled propofol conc. [ppb]
Marsh TCI predicted plasma conc. [µg/ml]
Plasma propofol conc. [µg/ml]
Pneumoperitoneum/Deflation
10
8
6
4
2
0
Concentration
0:00:00
1:00:00
2:00:00
Elapsed time (minutes)

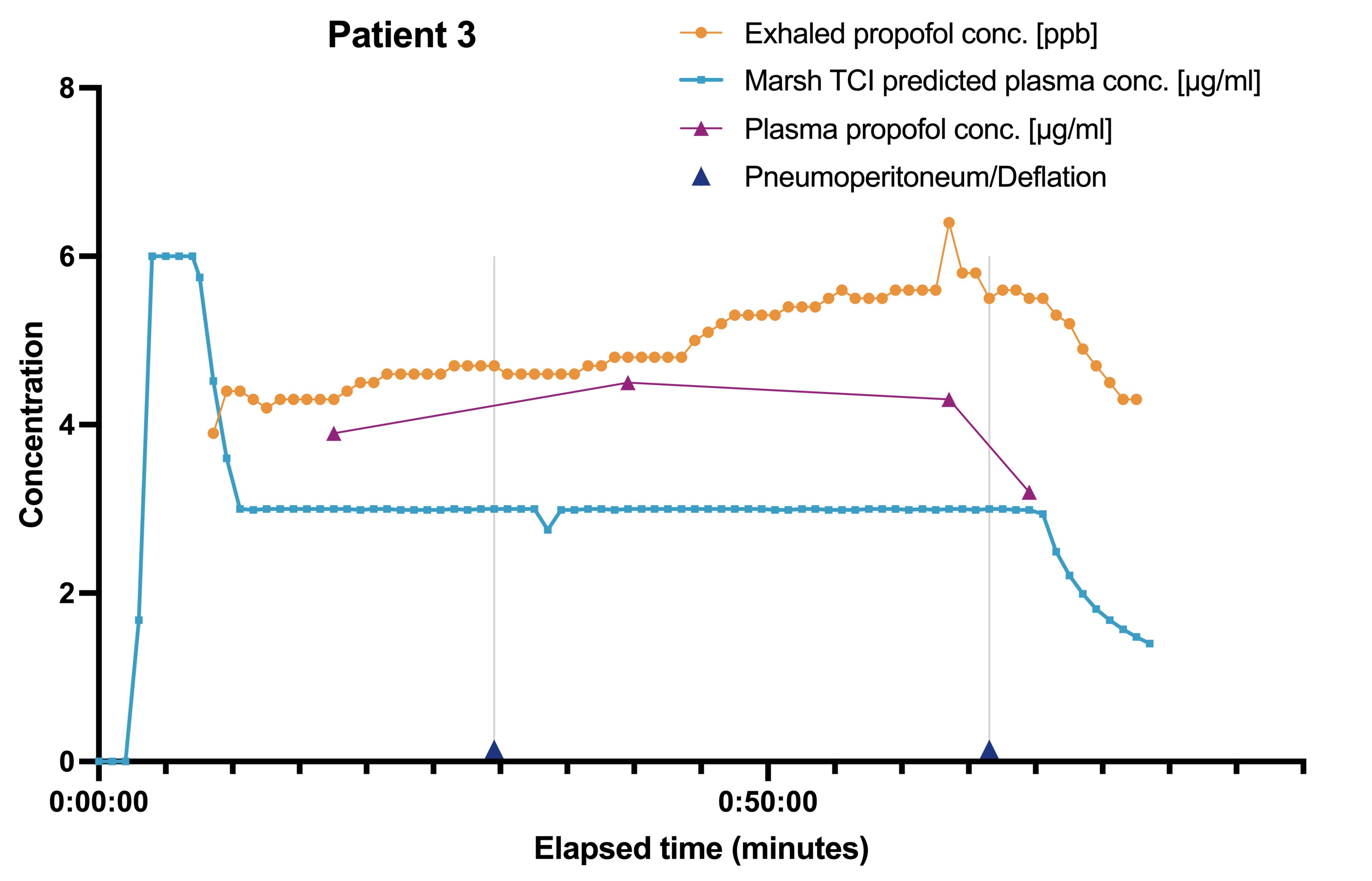

Patient 3
Exhaled propofol conc. [ppb]
Marsh TCI predicted plasma conc. [µg/ml]
Plasma propofol conc. [µg/ml]
Pneumoperitoneum/Deflation
Concentration
8
6
4
2
0
0:00:00
0:50:00
Elapsed time (minutes)

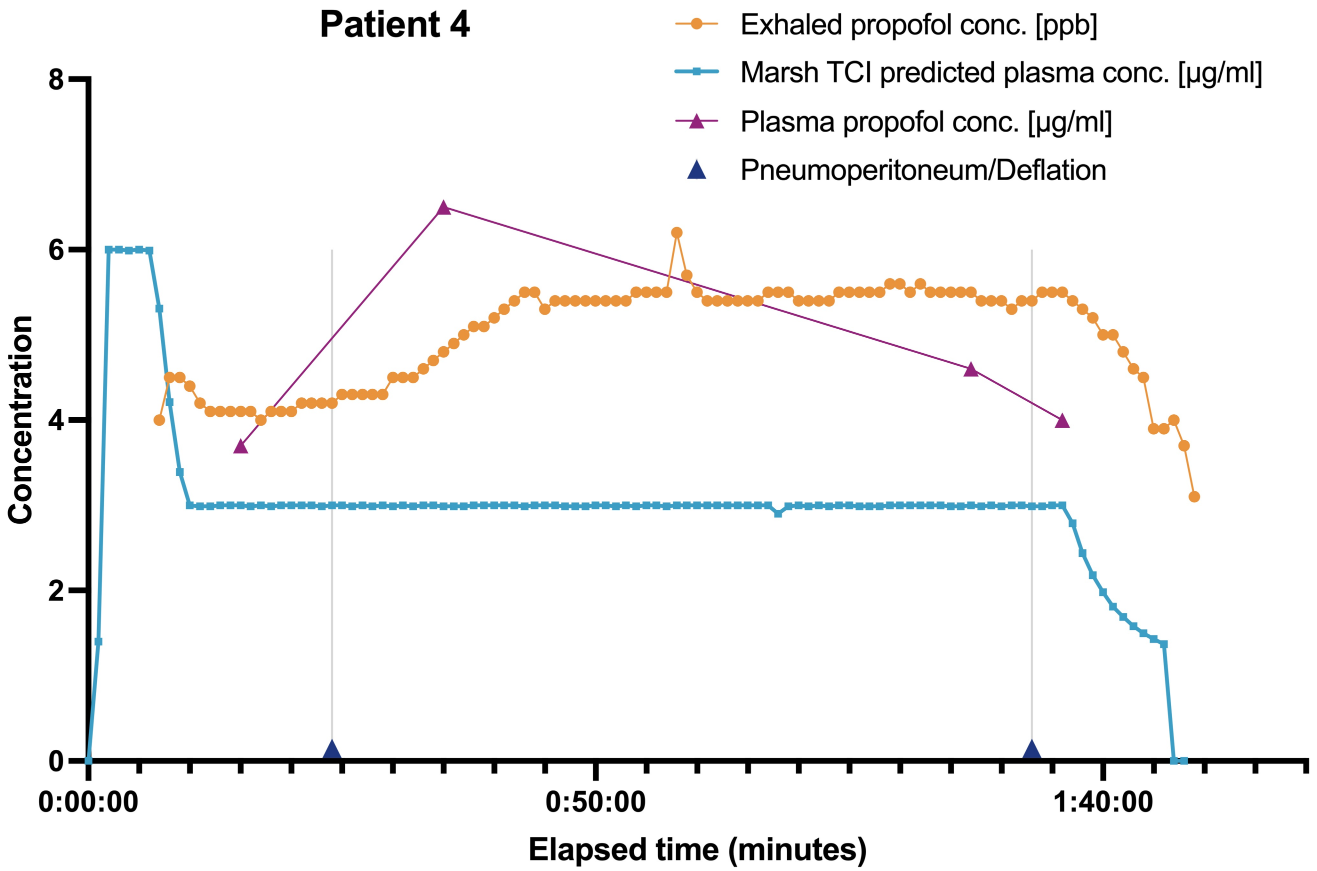

Patient 4
Exhaled propofol conc. [ppb]
Marsh TCI predicted plasma conc. [µg/ml]
Plasma propofol conc. [µg/ml]
Pneumoperitoneum/Deflation
8
6
4
2
0
Concentration
0:00:00
0:50:00
1:40:00
Elapsed time (minutes)

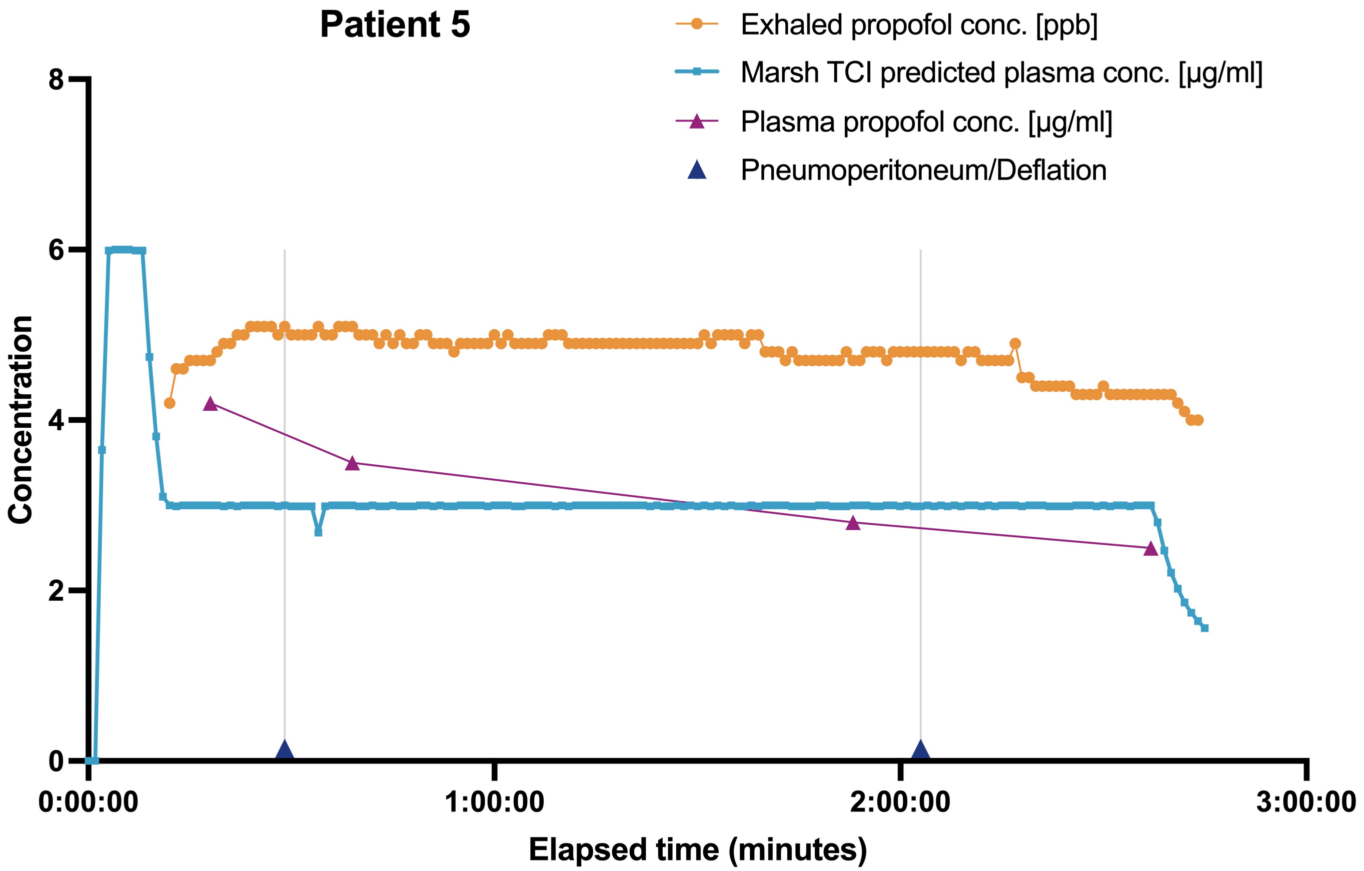

Patient 5
Exhaled propofol conc. [ppb]
Marsh TCI predicted plasma conc. [µg/ml]
Plasma propofol conc. [µg/ml]
Pneumoperitoneum/Deflation
Concentration
0
2
4
6
8
0:00:00
1:00:00
2:00:00
3:00:00
Elapsed time (minutes)

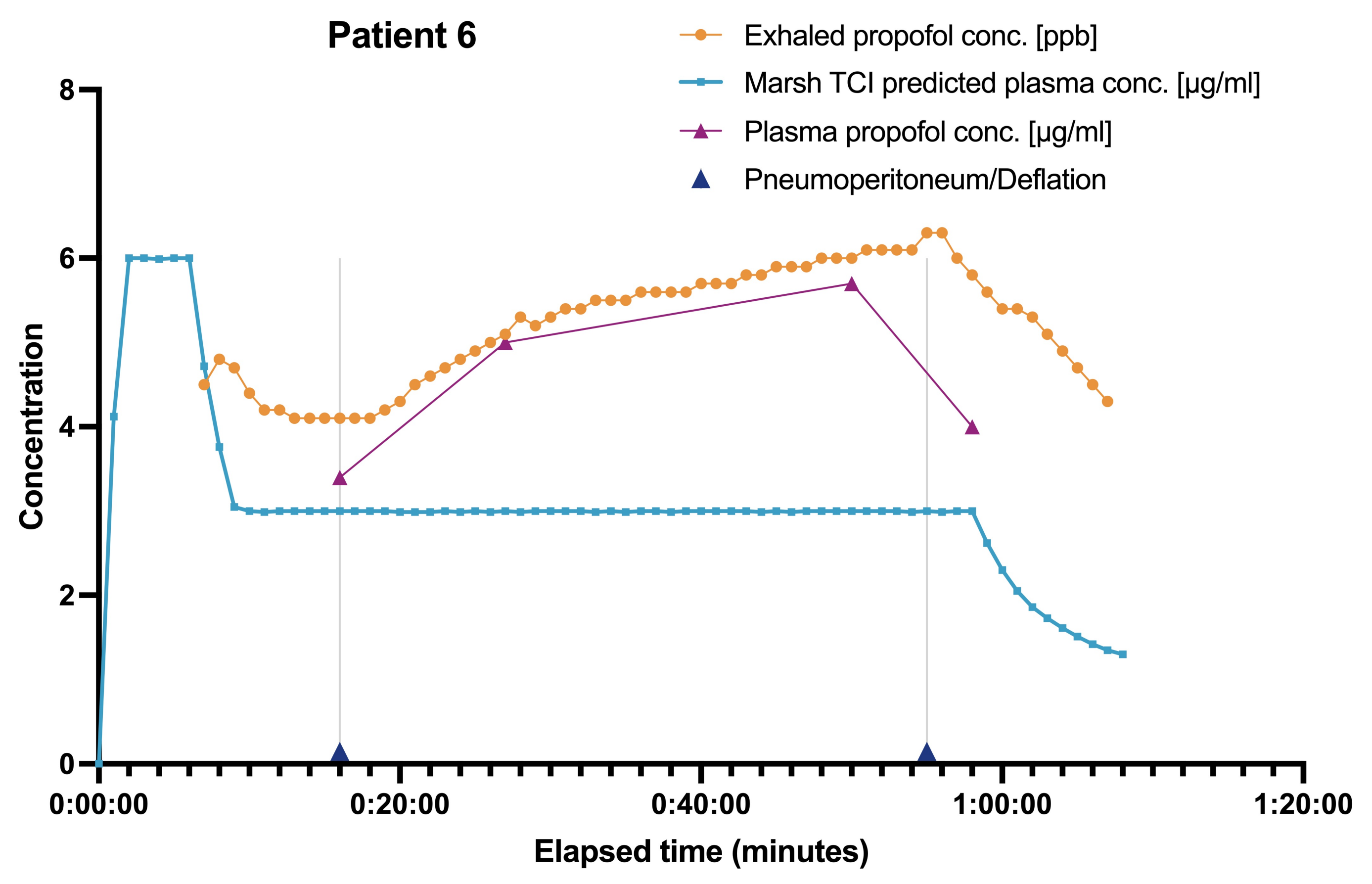

Patient 6
Exhaled propofol conc. [ppb]
Marsh TCI predicted plasma conc. [µg/ml]
Plasma propofol conc. [µg/ml]
Pneumoperitoneum/Deflation
Concentration
0
2
4
6
8
0:00:00
0:20:00
0:40:00
1:00:00
1:20:00
Elapsed time (minutes)

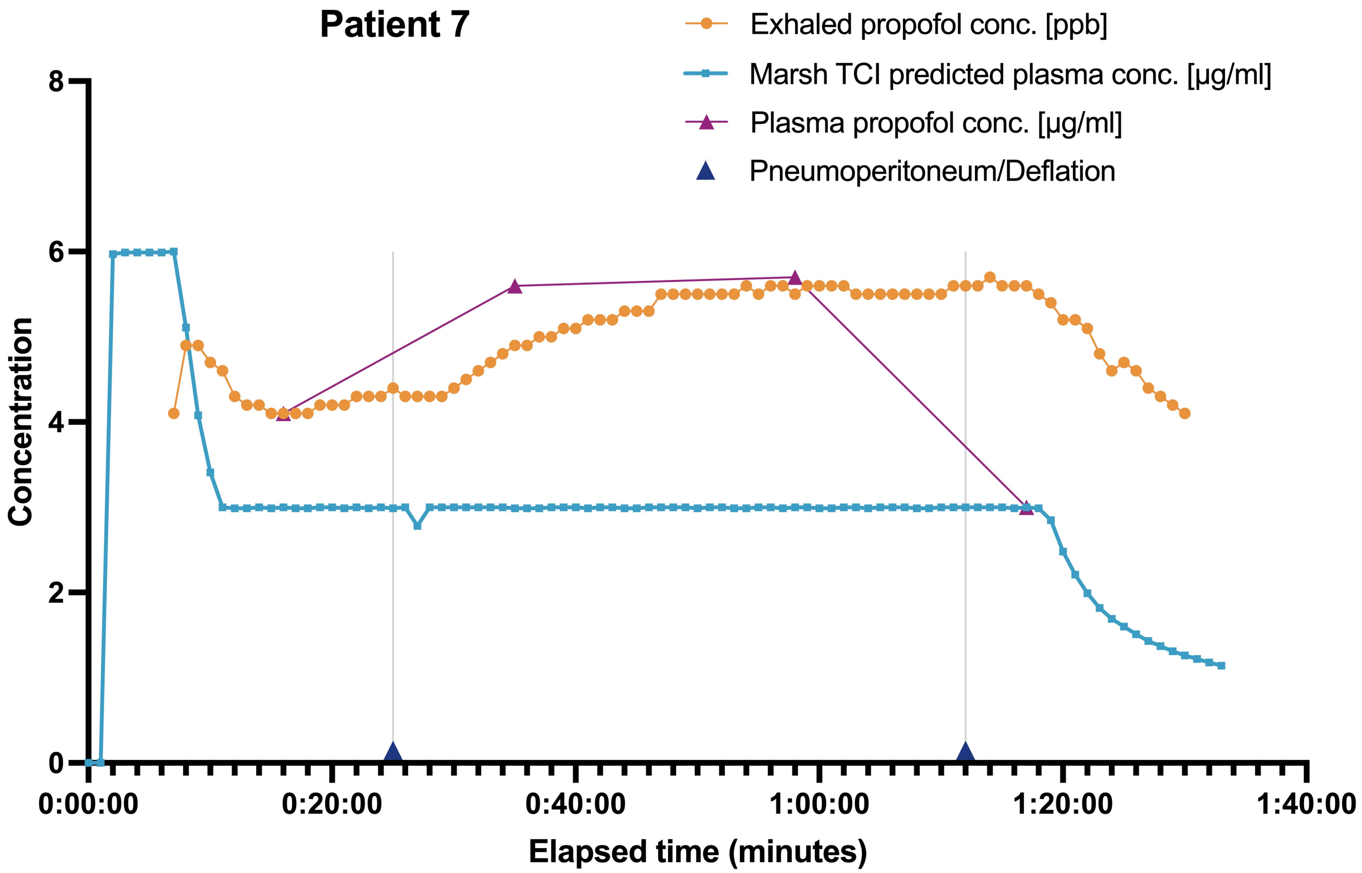

Patient 7
Exhaled propofol conc. [ppb]
Marsh TCI predicted plasma conc. [μg/ml]
Plasma propofol conc. [μg/ml]
Pneumoperitoneum/Deflation
Concentration
8
6
4
2
0
0:00:00
0:20:00
0:40:00
1:00:00
1:20:00
1:40:00
Elapsed time (minutes)

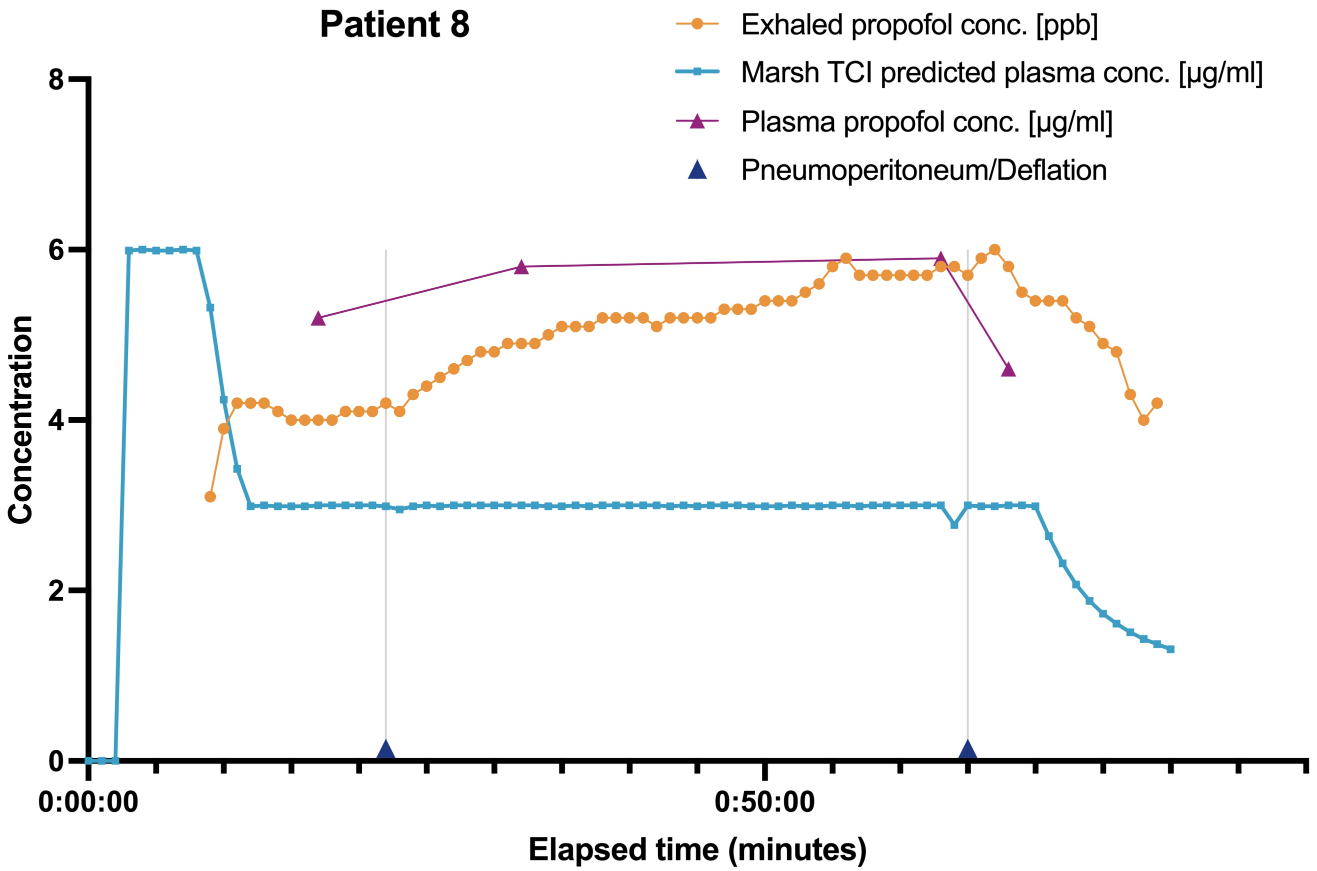
Patient 8
Exhaled propofol conc. [ppb]
Marsh TCI predicted plasma conc. [μg/ml]
Plasma propofol conc. [μg/ml]
Pneumoperitoneum/Deflation
Concentration
8
6
4
2
0
0:00:00
0:50:00
Elapsed time (minutes)

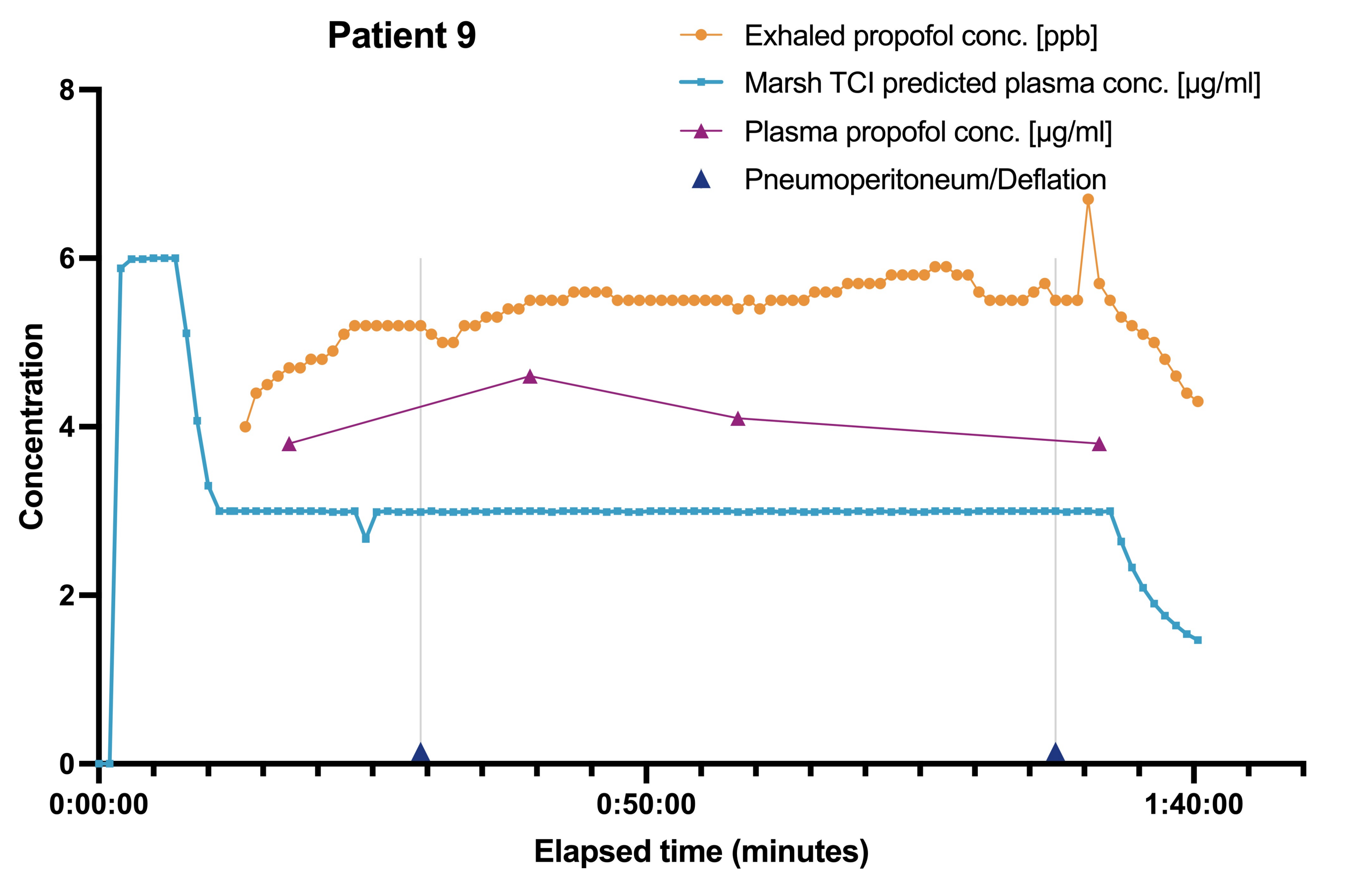
Patient 9
Exhaled propofol conc. [ppb]
Marsh TCI predicted plasma conc. [µg/ml]
Plasma propofol conc. [µg/ml]
Pneumoperitoneum/Deflation
8
6
4
2
0
Concentration
0:00:00
0:50:00
1:40:00
Elapsed time (minutes)

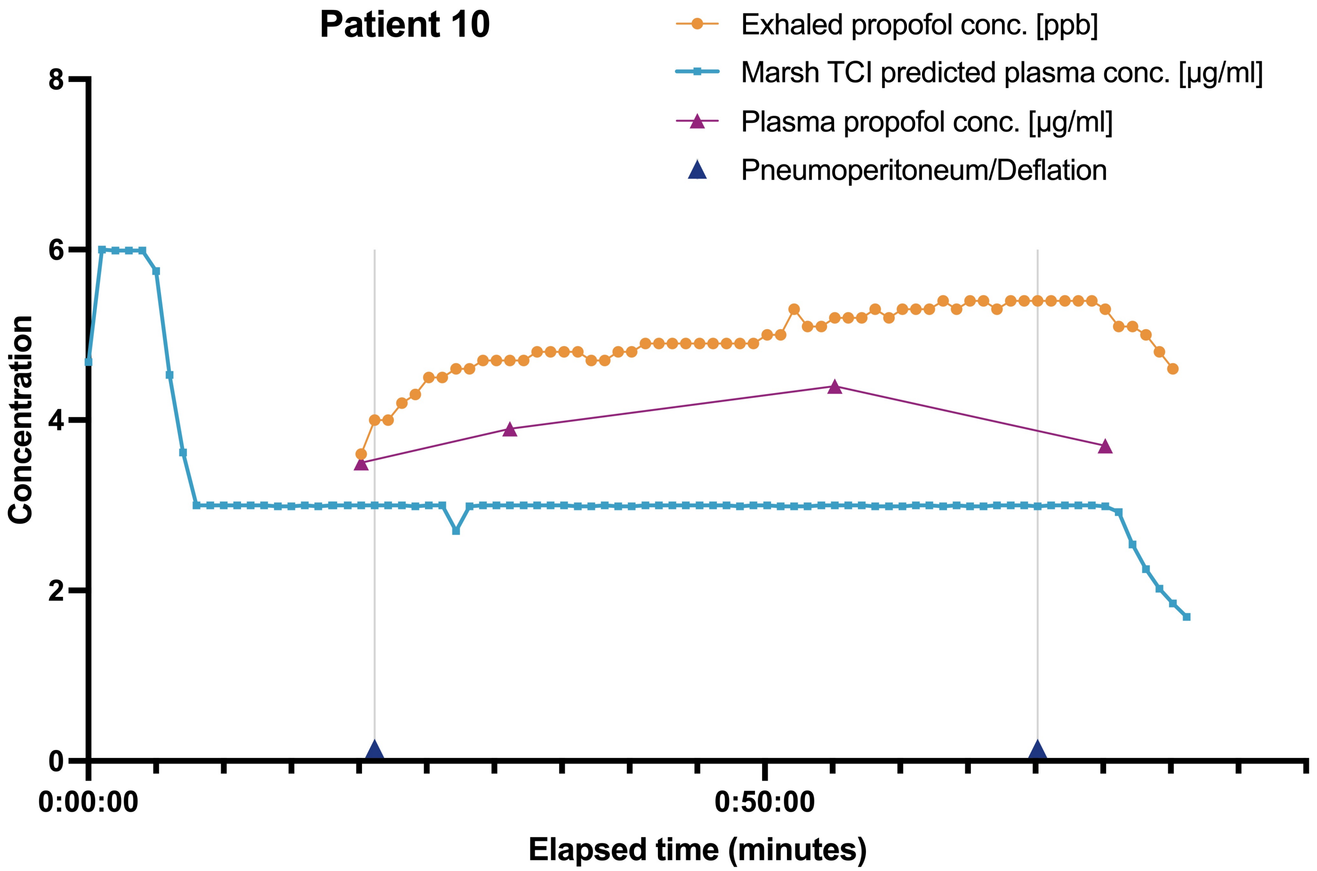

Patient 10
Exhaled propofol conc. [ppb]
Marsh TCI predicted plasma conc. [µg/ml]
Plasma propofol conc. [µg/ml]
Pneumoperitoneum/Deflation
Concentration
8
6
4
2
0
0:00:00
0:50:00
Elapsed time (minutes)

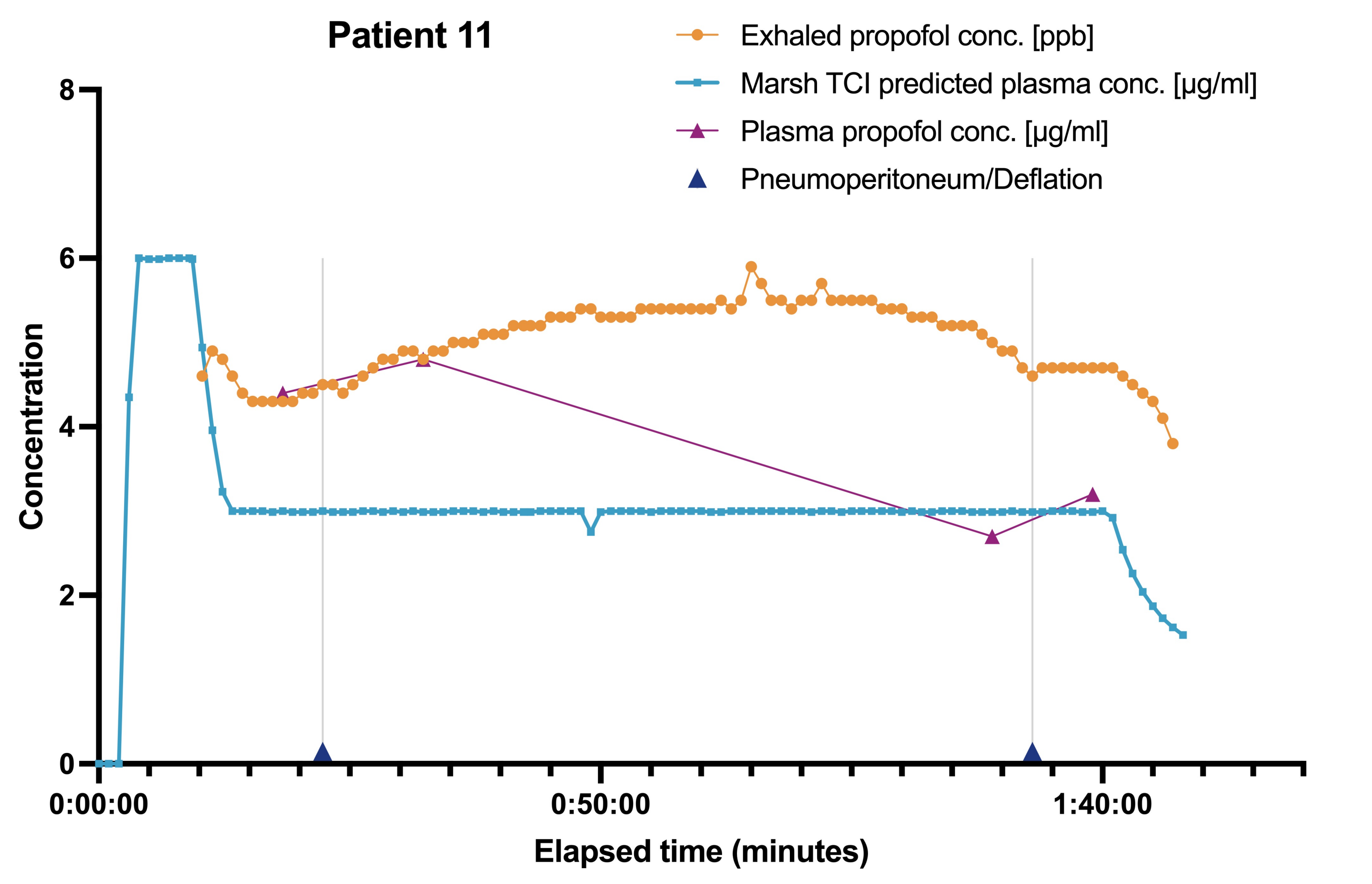

Patient 11
Exhaled propofol conc. [ppb]
Marsh TCI predicted plasma conc. [µg/ml]
Plasma propofol conc. [µg/ml]
Pneumoperitoneum/Deflation
Concentration
8
6
4
2
0
0:00:00
0:50:00
1:40:00
Elapsed time (minutes)

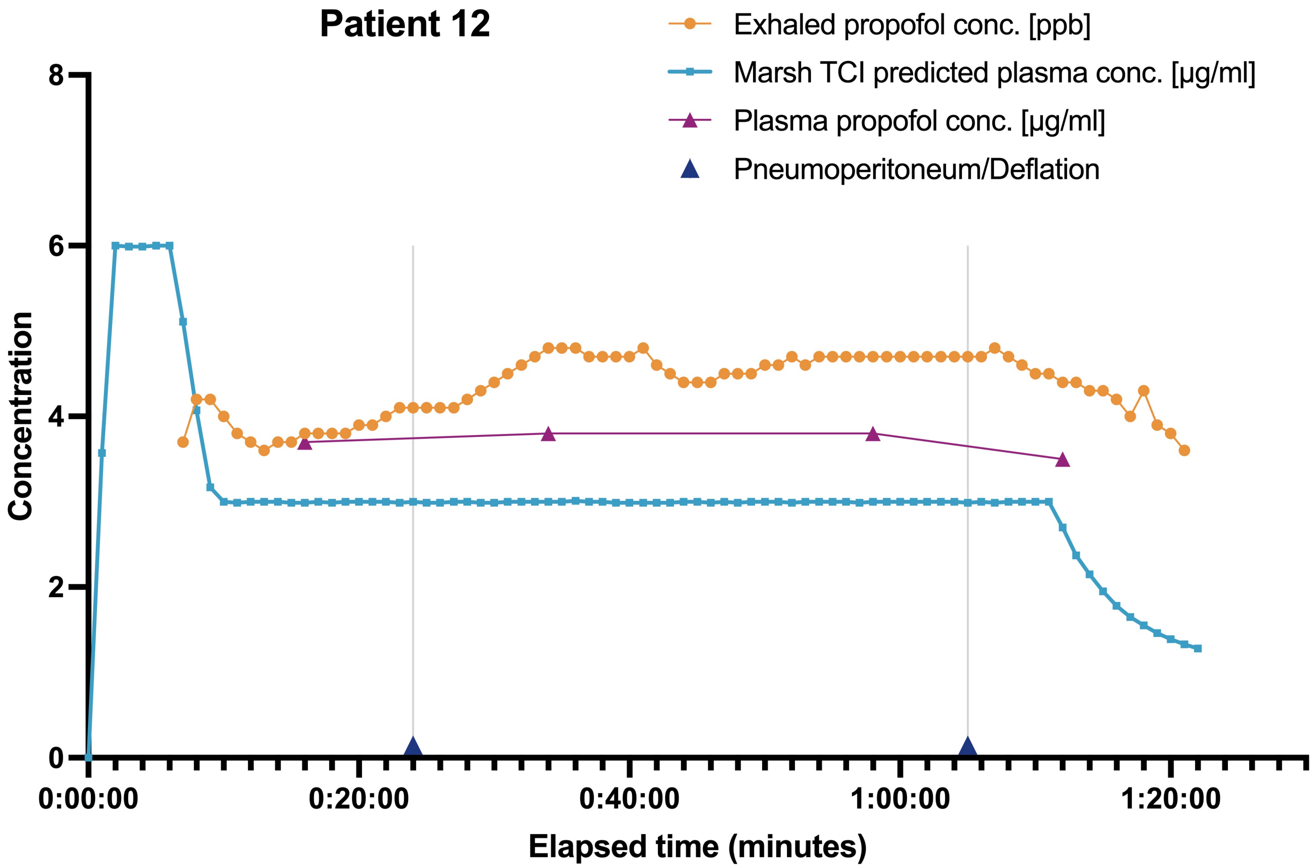

Patient 12
Exhaled propofol conc. [ppb]
Marsh TCI predicted plasma conc. [µg/ml]
Plasma propofol conc. [µg/ml]
Pneumoperitoneum/Deflation
Concentration
0
2
4
6
8
0:00:00
0:20:00
0:40:00
1:00:00
1:20:00
Elapsed time (minutes)

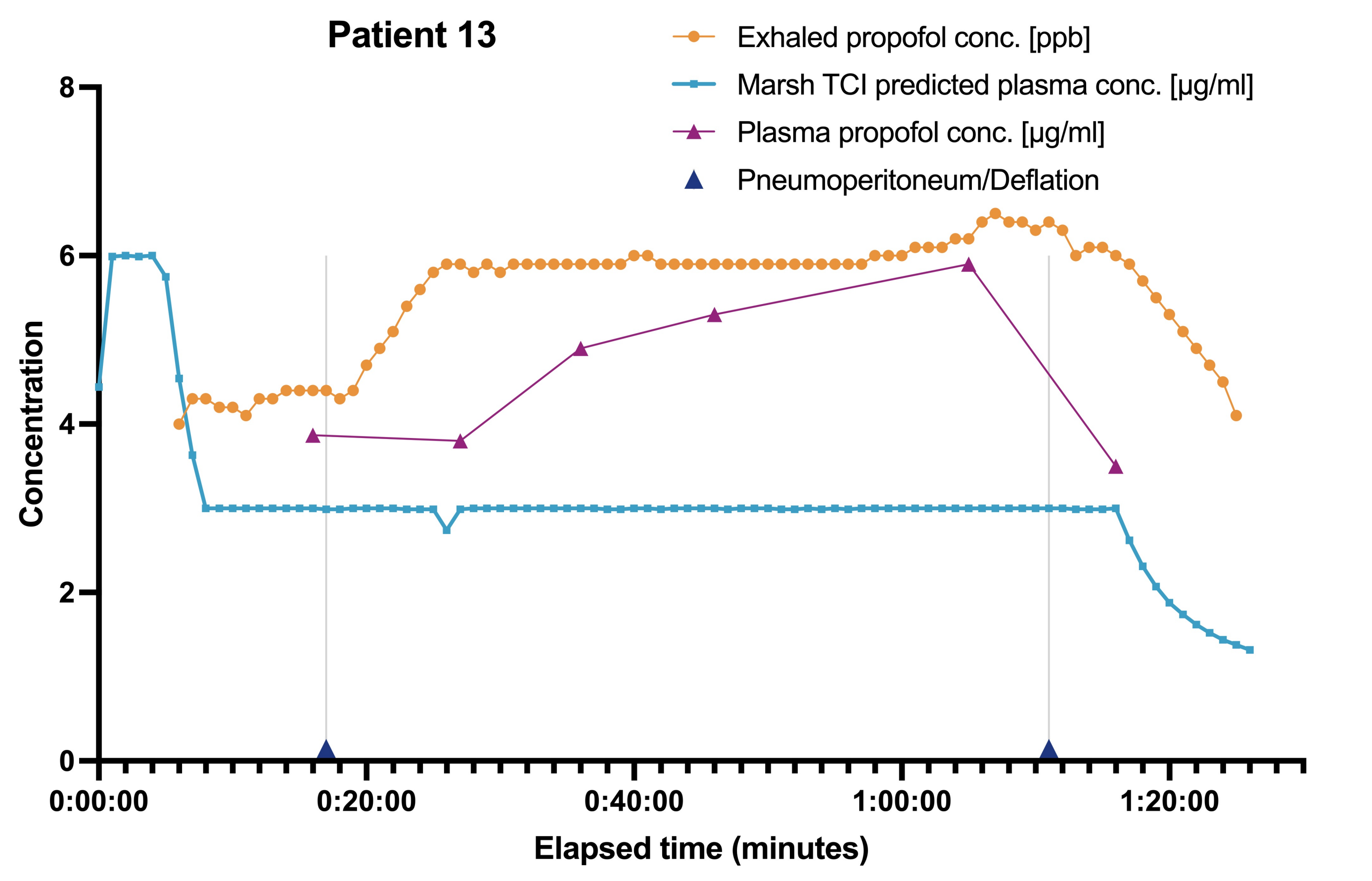
Patient 13
Exhaled propofol conc. [ppb]
Marsh TCI predicted plasma conc. [μg/ml]
Plasma propofol conc. [μg/ml]
Pneumoperitoneum/Deflation
Concentration
0
2
4
6
8
0:00:00
0:20:00
0:40:00
1:00:00
1:20:00
Elapsed time (minutes)

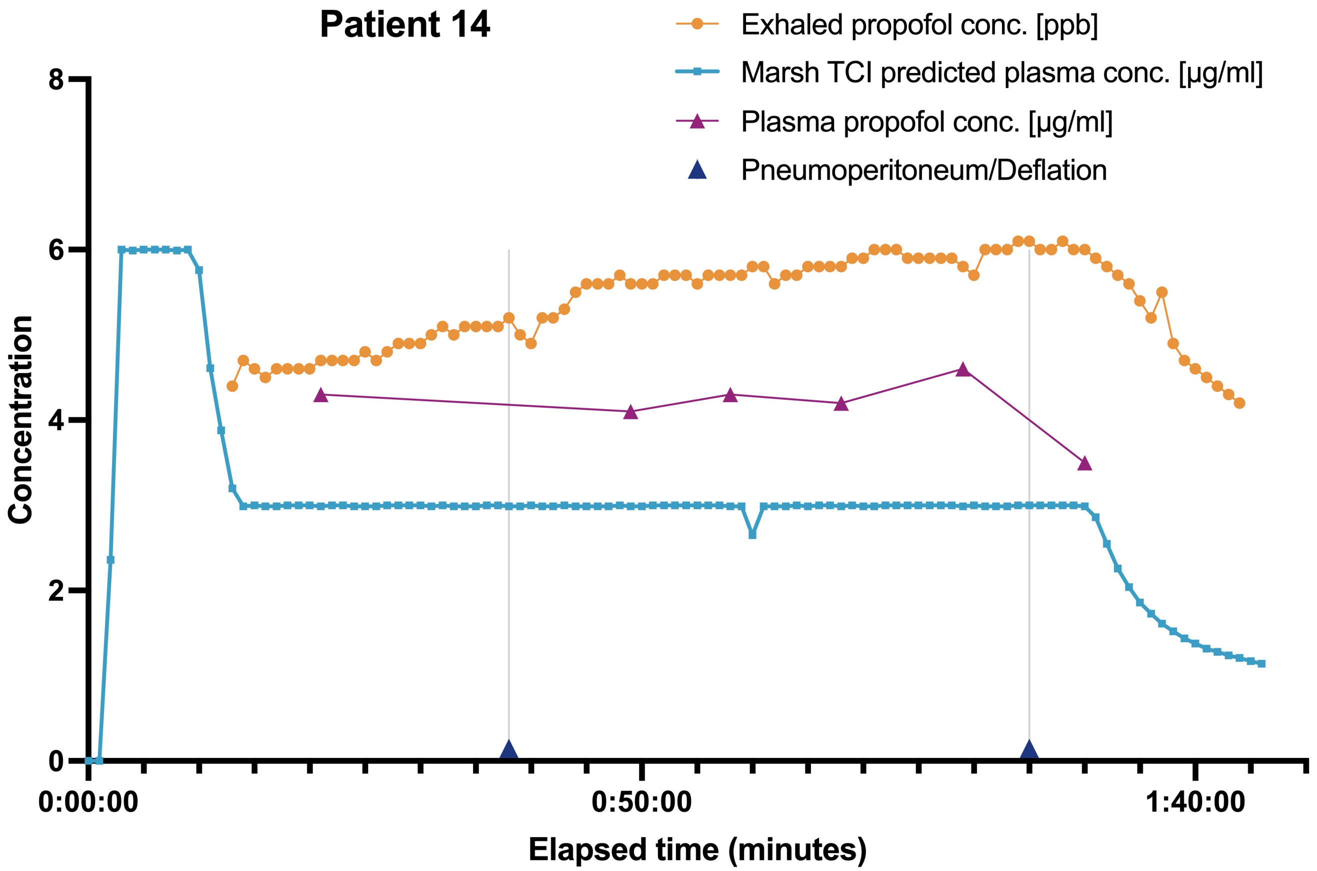

Patient 14
Exhaled propofol conc. [ppb]
Marsh TCI predicted plasma conc. [µg/ml]
Plasma propofol conc. [µg/ml]
Pneumoperitoneum/Deflation
Concentration
8
6
4
2
0
0:00:00
0:50:00
1:40:00
Elapsed time (minutes)

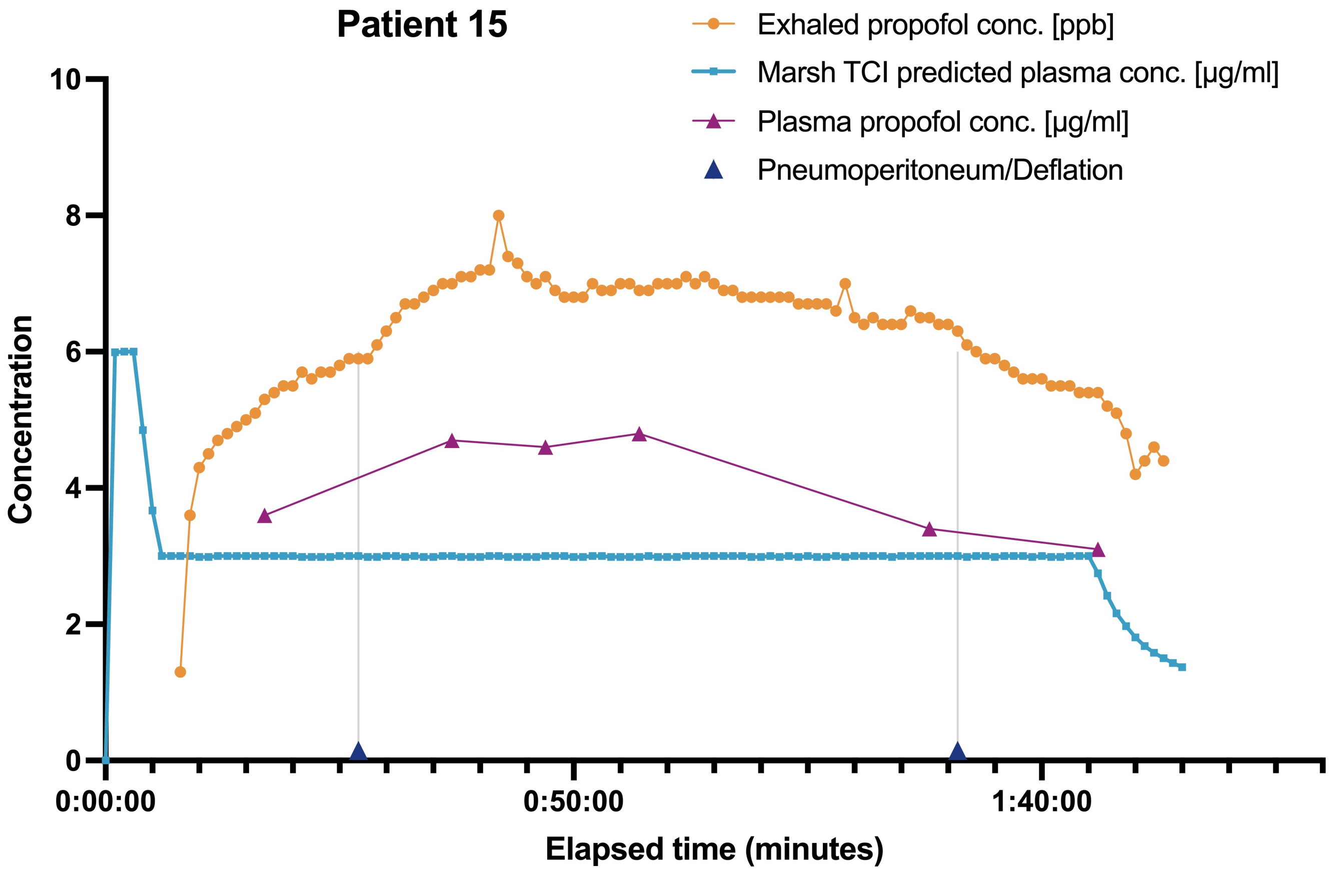

Patient 15
Exhaled propofol conc. [ppb]
Marsh TCI predicted plasma conc. [µg/ml]
Plasma propofol conc. [µg/ml]
Pneumoperitoneum/Deflation
Concentration
10
8
6
4
2
0
0:00:00
0:50:00
1:40:00
Elapsed time (minutes)

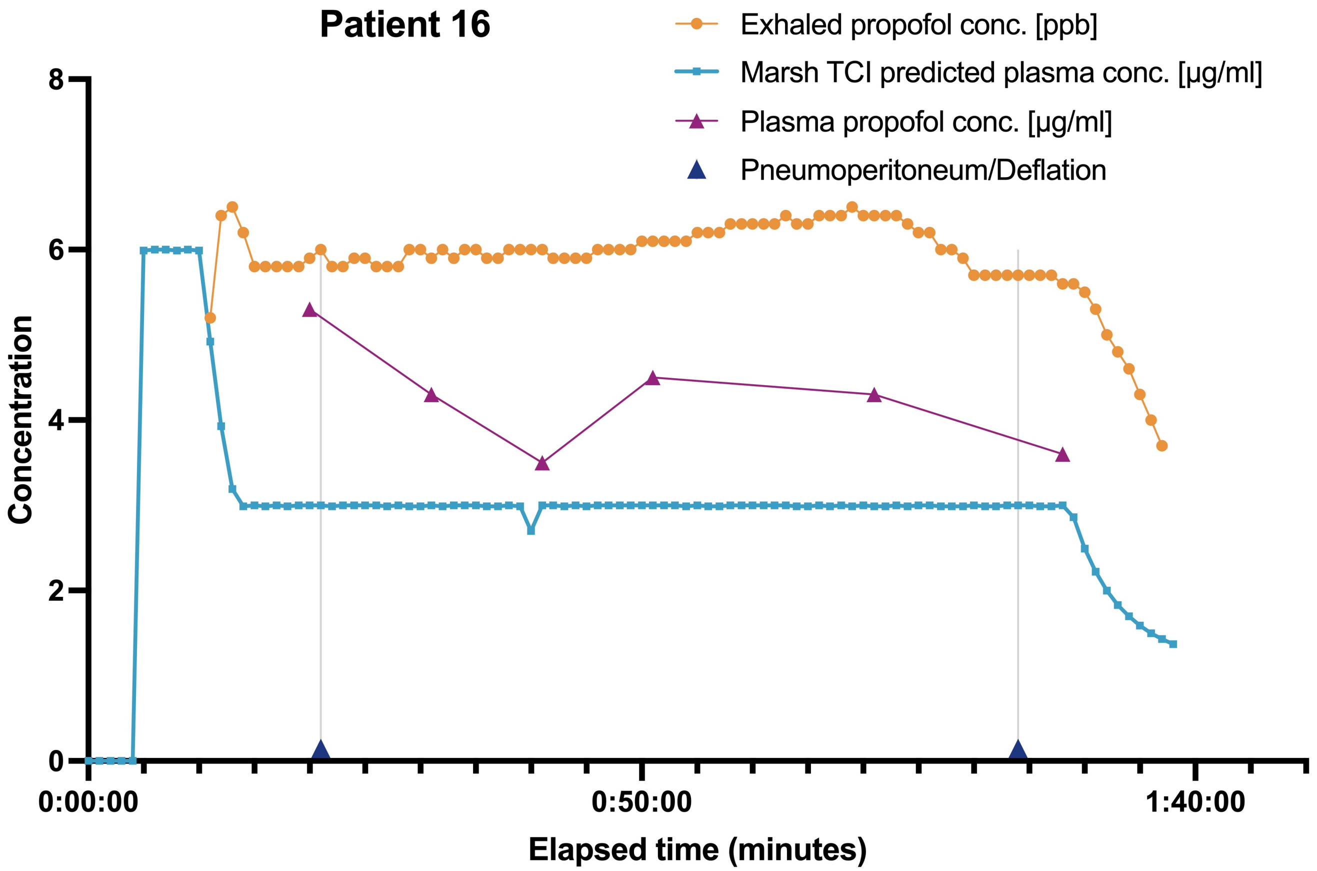
Patient 16
Exhaled propofol conc. [ppb]
Marsh TCI predicted plasma conc. [µg/ml]
Plasma propofol conc. [µg/ml]
Pneumoperitoneum/Deflation
Concentration
8
6
4
2
0
0:00:00
0:50:00
1:40:00
Elapsed time (minutes)

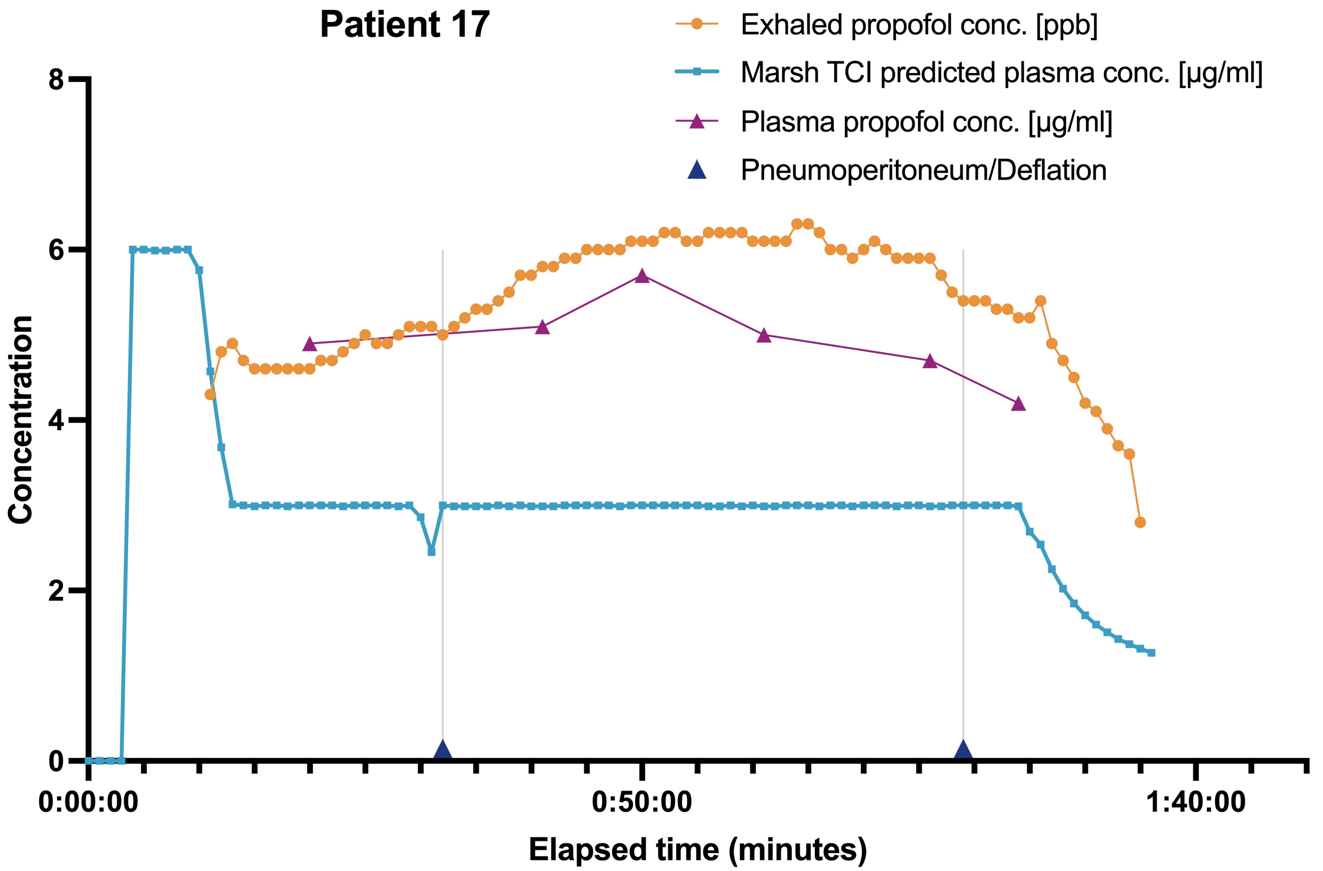
Patient 17
Exhaled propofol conc. [ppb]
Marsh TCI predicted plasma conc. [µg/ml]
Plasma propofol conc. [µg/ml]
Pneumoperitoneum/Deflation
Concentration
8
6
4
2
0
0:00:00
0:50:00
1:40:00
Elapsed time (minutes)

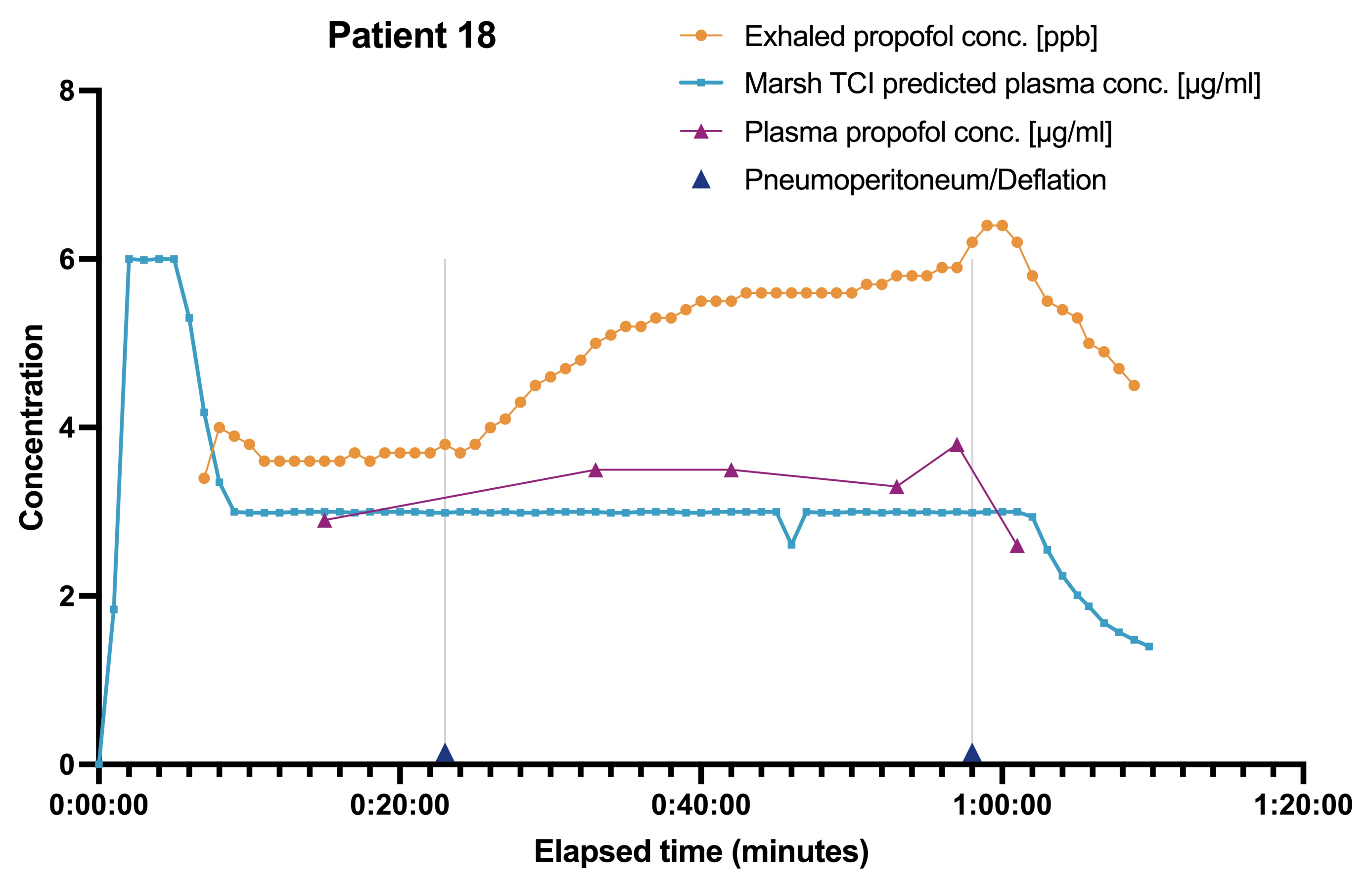

Patient 18
Exhaled propofol conc. [ppb]
Marsh TCI predicted plasma conc. [µg/ml]
Plasma propofol conc. [µg/ml]
Pneumoperitoneum/Deflation
Concentration
0
2
4
6
8
0:00:00
0:20:00
0:40:00
1:00:00
1:20:00
Elapsed time (minutes)

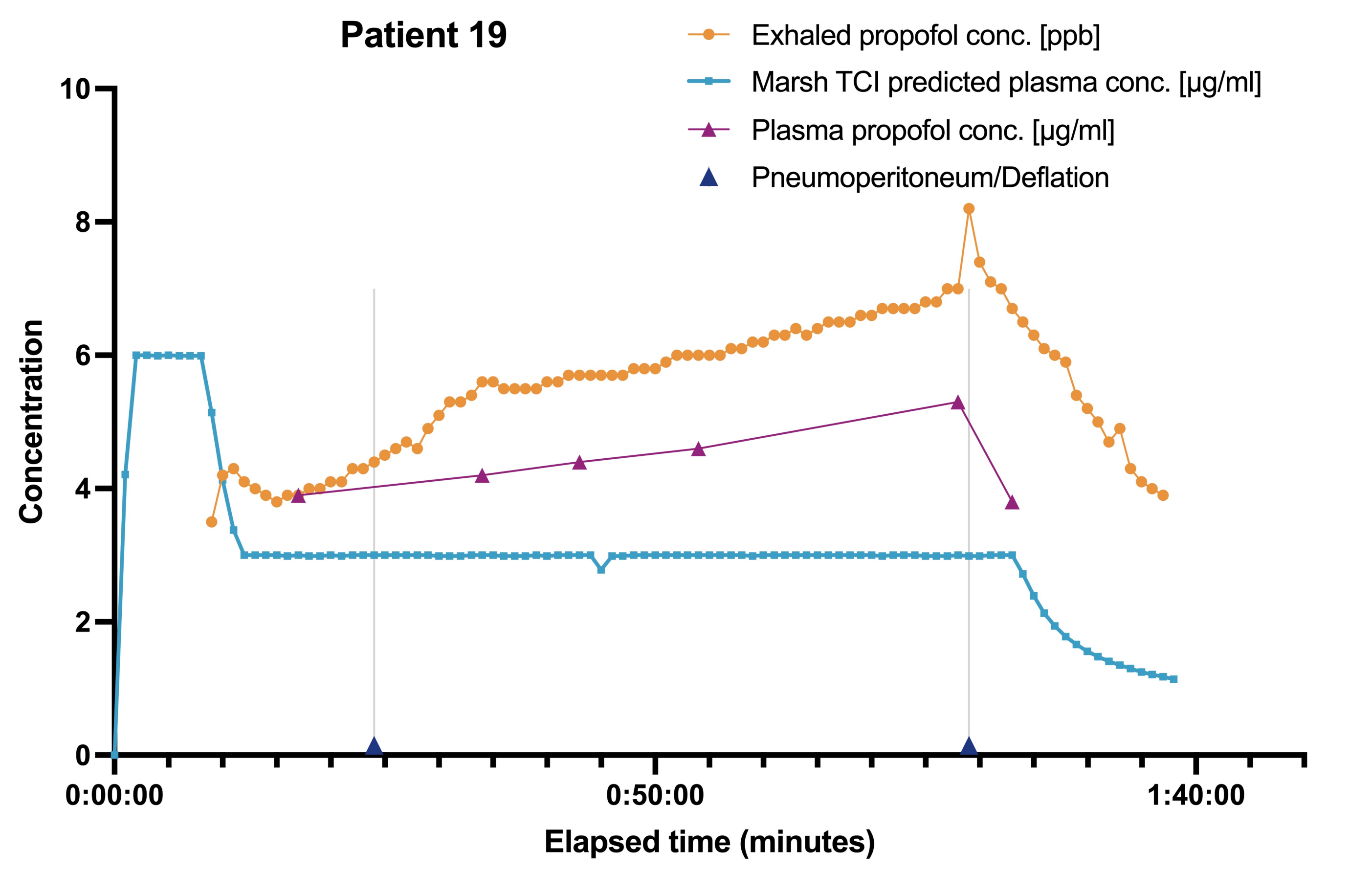

Patient 19
Exhaled propofol conc. [ppb]
Marsh TCI predicted plasma conc. [µg/ml]
Plasma propofol conc. [µg/ml]
Pneumoperitoneum/Deflation
Concentration
10
8
6
4
2
0
0:00:00
0:50:00
1:40:00
Elapsed time (minutes)

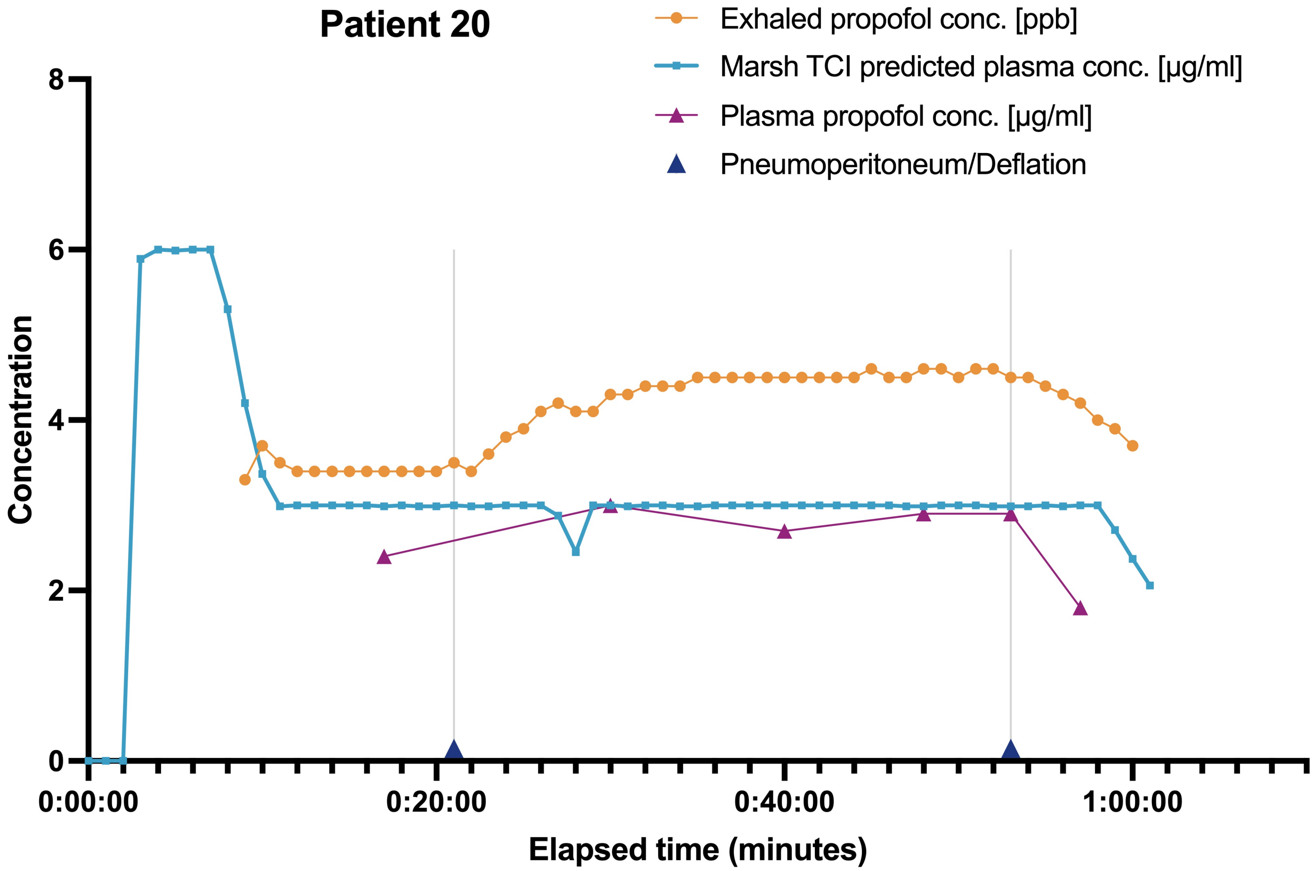

Patient 20
Exhaled propofol conc. [ppb]
Marsh TCI predicted plasma conc. [µg/ml]
Plasma propofol conc. [µg/ml]
Pneumoperitoneum/Deflation
Concentration
8
6
4
2
0
0:00:00
0:20:00
0:40:00
1:00:00
Elapsed time (minutes)

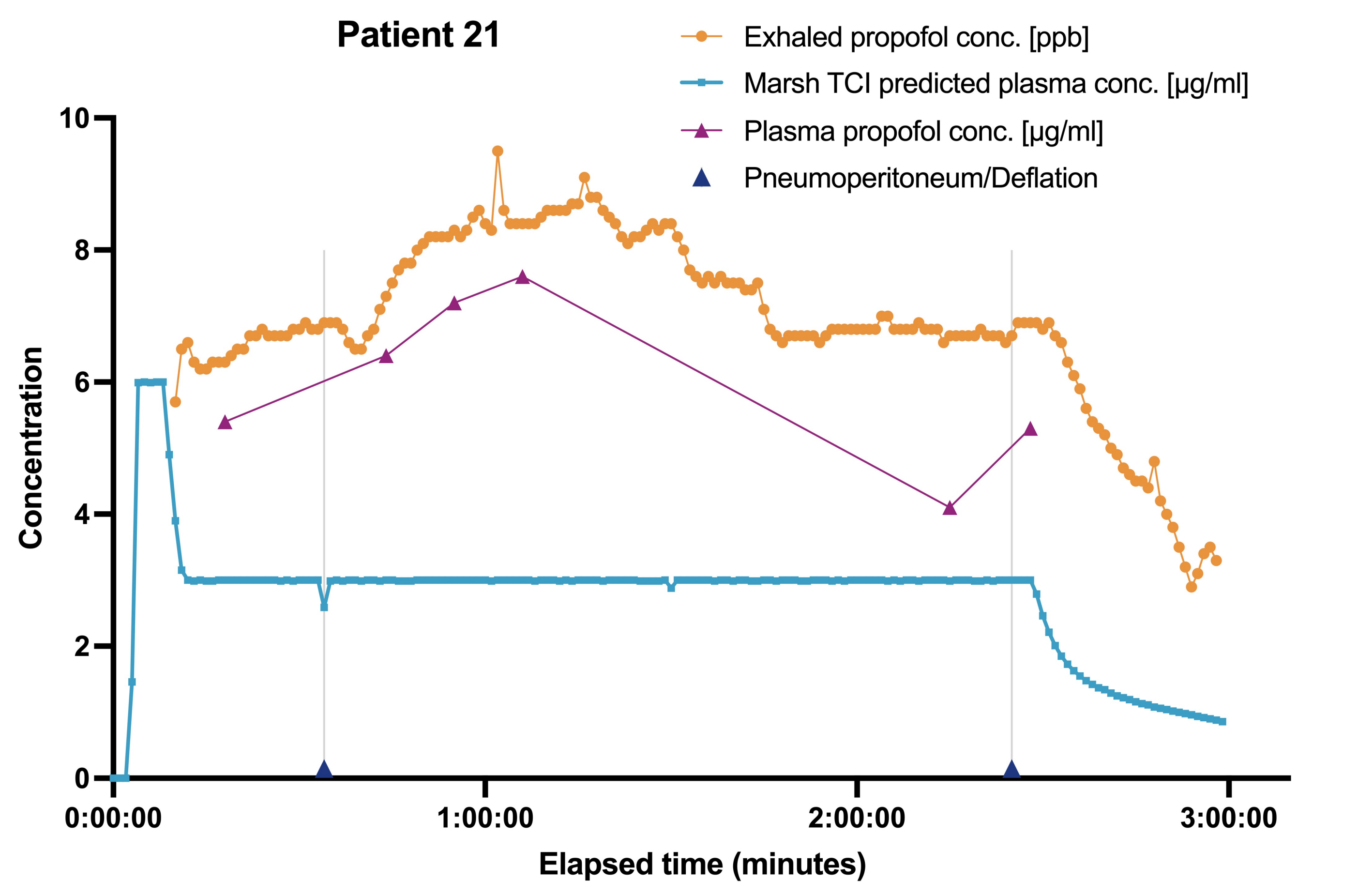

Patient 21
Exhaled propofol conc. [ppb]
Marsh TCI predicted plasma conc. [µg/ml]
Plasma propofol conc. [µg/ml]
Pneumoperitoneum/Deflation
Concentration
10
8
6
4
2
0
0:00:00
1:00:00
2:00:00
3:00:00
Elapsed time (minutes)

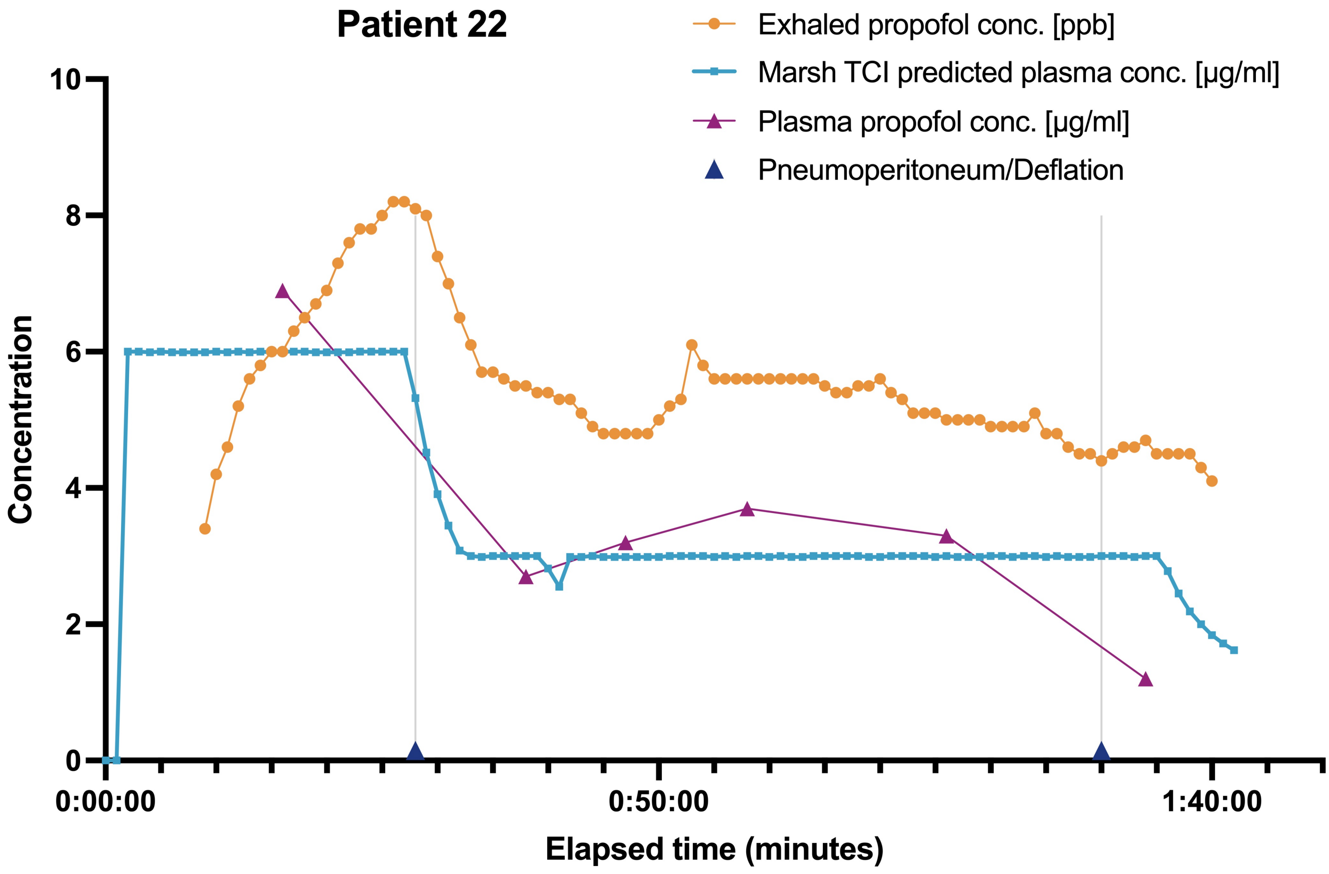

Patient 22
Exhaled propofol conc. [ppb]
Marsh TCI predicted plasma conc. [µg/ml]
Plasma propofol conc. [µg/ml]
Pneumoperitoneum/Deflation
Concentration
10
8
6
4
2
0
0:00:00
0:50:00
1:40:00
Elapsed time (minutes)

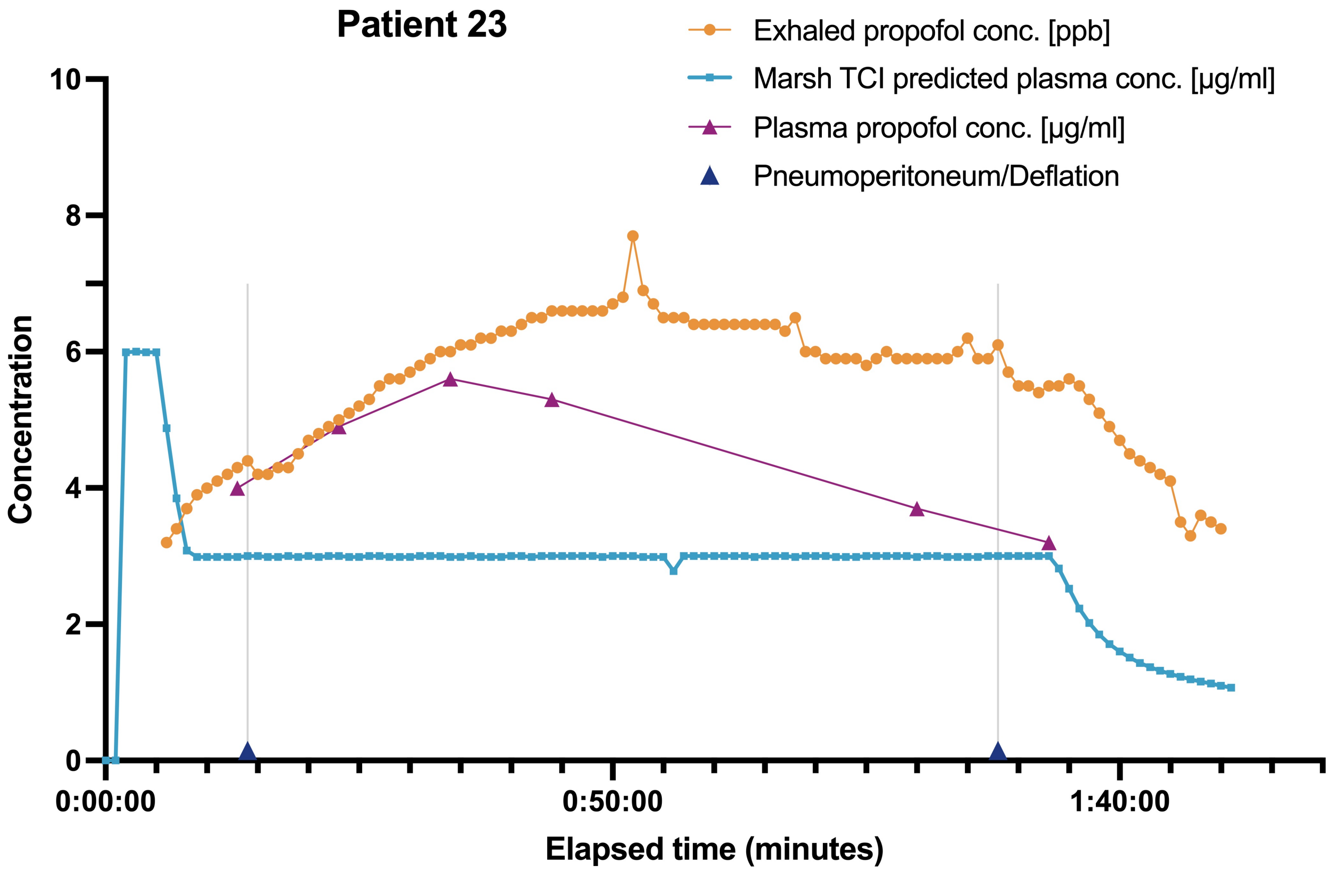

Patient 23
Exhaled propofol conc. [ppb]
Marsh TCI predicted plasma conc. [µg/ml]
Plasma propofol conc. [µg/ml]
Pneumoperitoneum/Deflation
Concentration
10
8
6
4
2
0
0:00:00
0:50:00
1:40:00
Elapsed time (minutes)

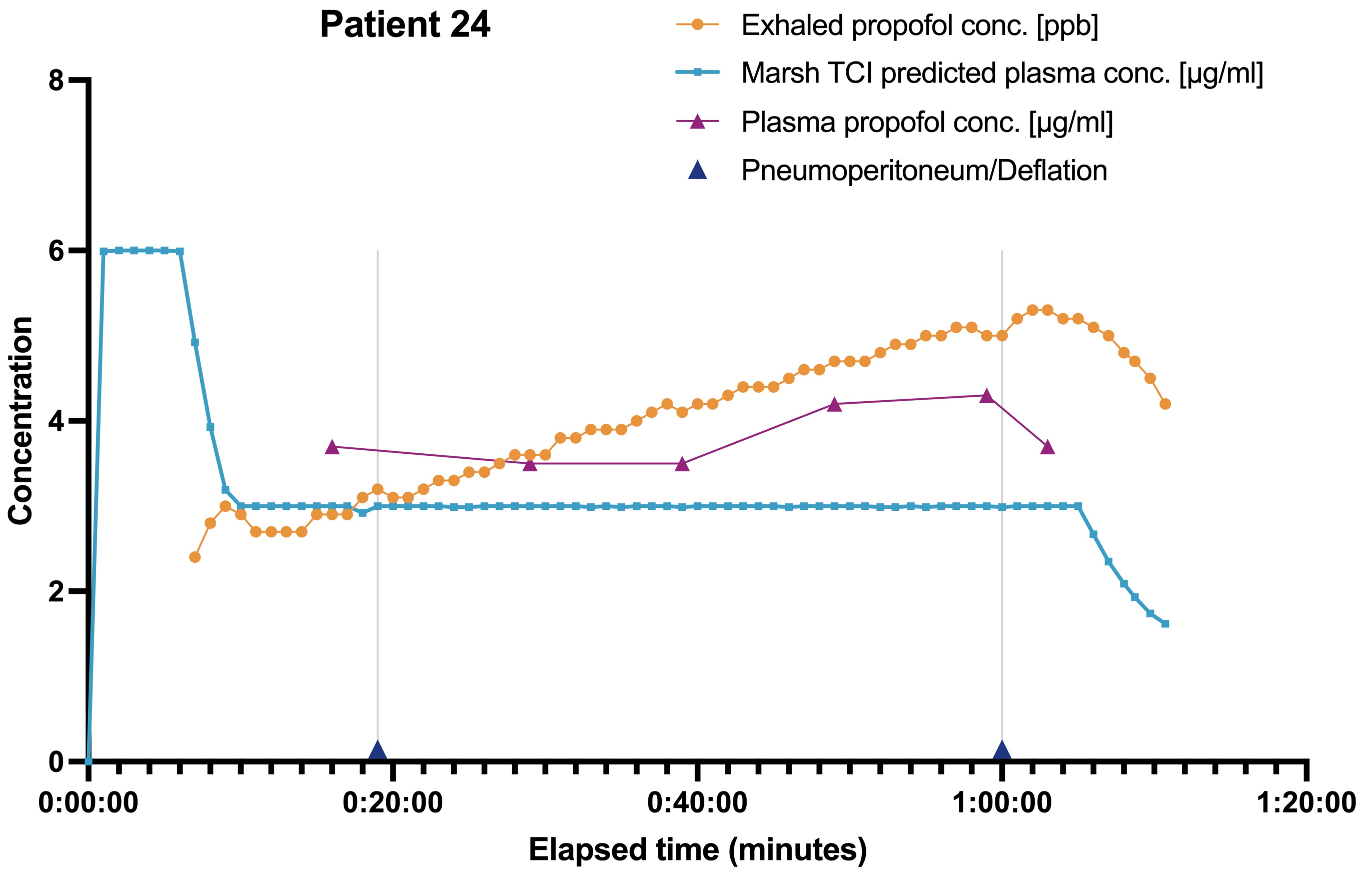

Patient 24
Exhaled propofol conc. [ppb]
Marsh TCI predicted plasma conc. [µg/ml]
Plasma propofol conc. [µg/ml]
Pneumoperitoneum/Deflation
Concentration
8
6
4
2
0
0:00:00
0:20:00
0:40:00
1:00:00
1:20:00
Elapsed time (minutes)

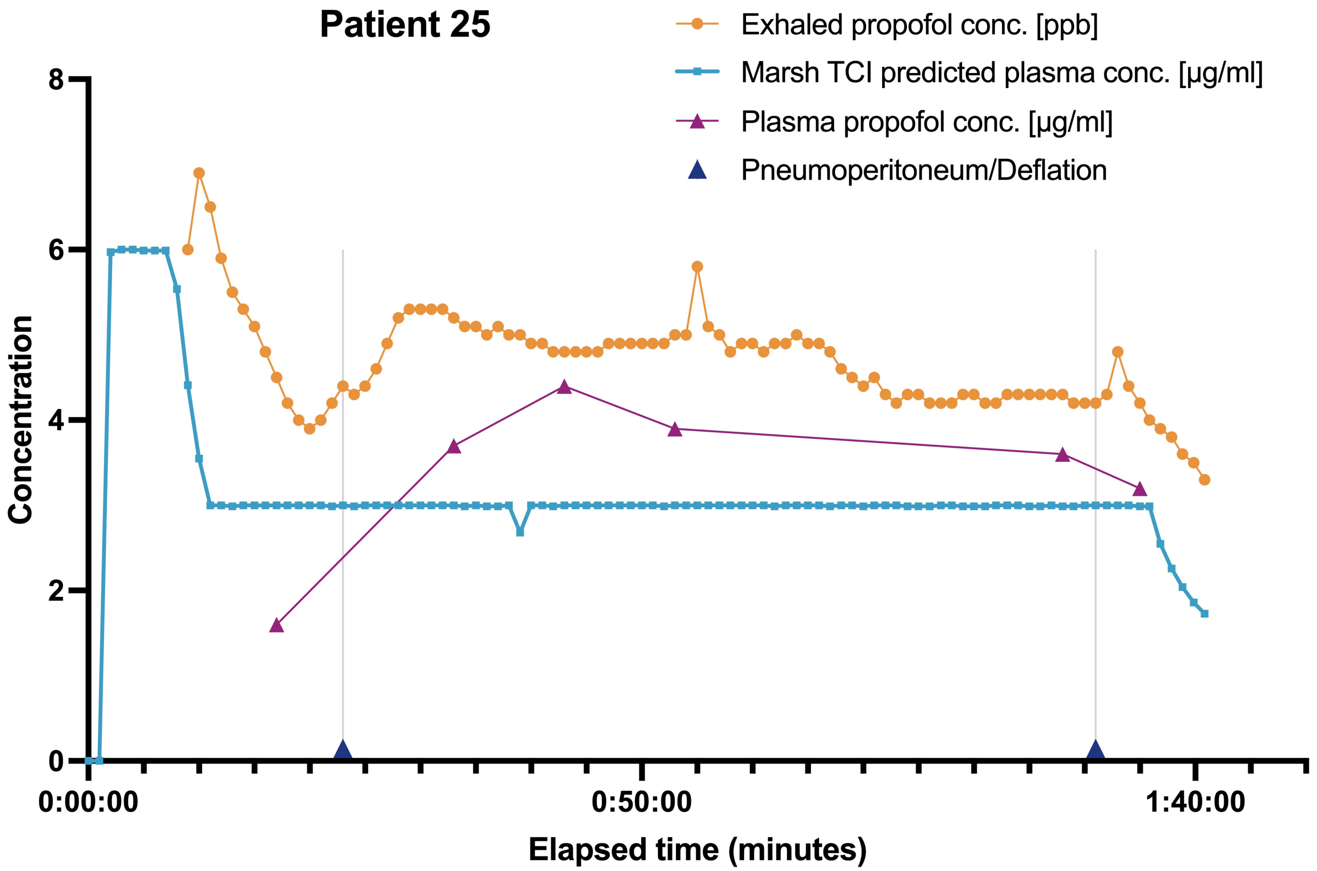

Patient 25
Exhaled propofol conc. [ppb]
Marsh TCI predicted plasma conc. [µg/ml]
Plasma propofol conc. [µg/ml]
Pneumoperitoneum/Deflation
Concentration
8
6
4
2
0
0:00:00
0:50:00
1:40:00
Elapsed time (minutes)

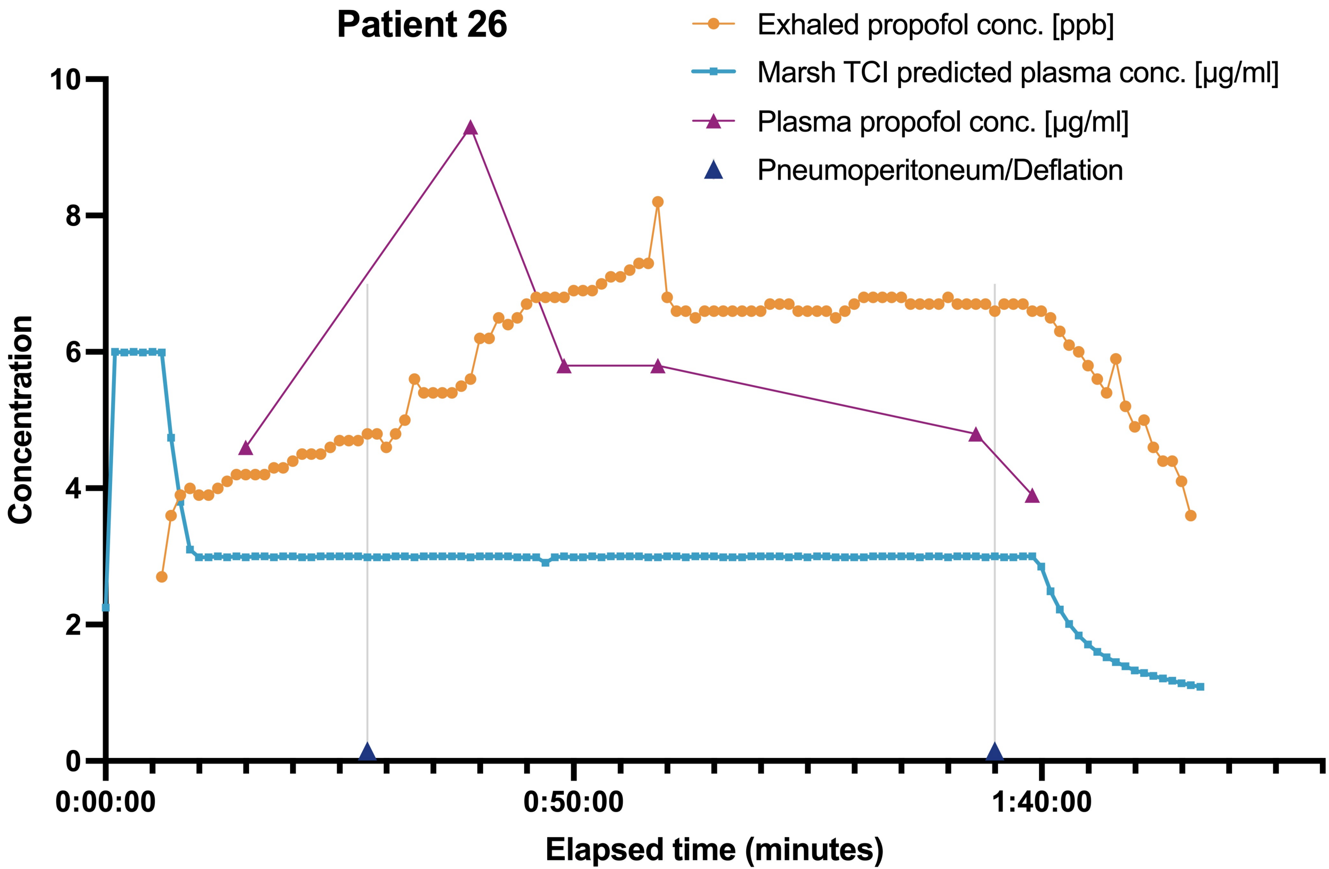
Patient 26
Exhaled propofol conc. [ppb]
Marsh TCI predicted plasma conc. [µg/ml]
Plasma propofol conc. [µg/ml]
Pneumoperitoneum/Deflation
Concentration
0
2
4
6
8
10
0:00:00
0:50:00
1:40:00
Elapsed time (minutes)

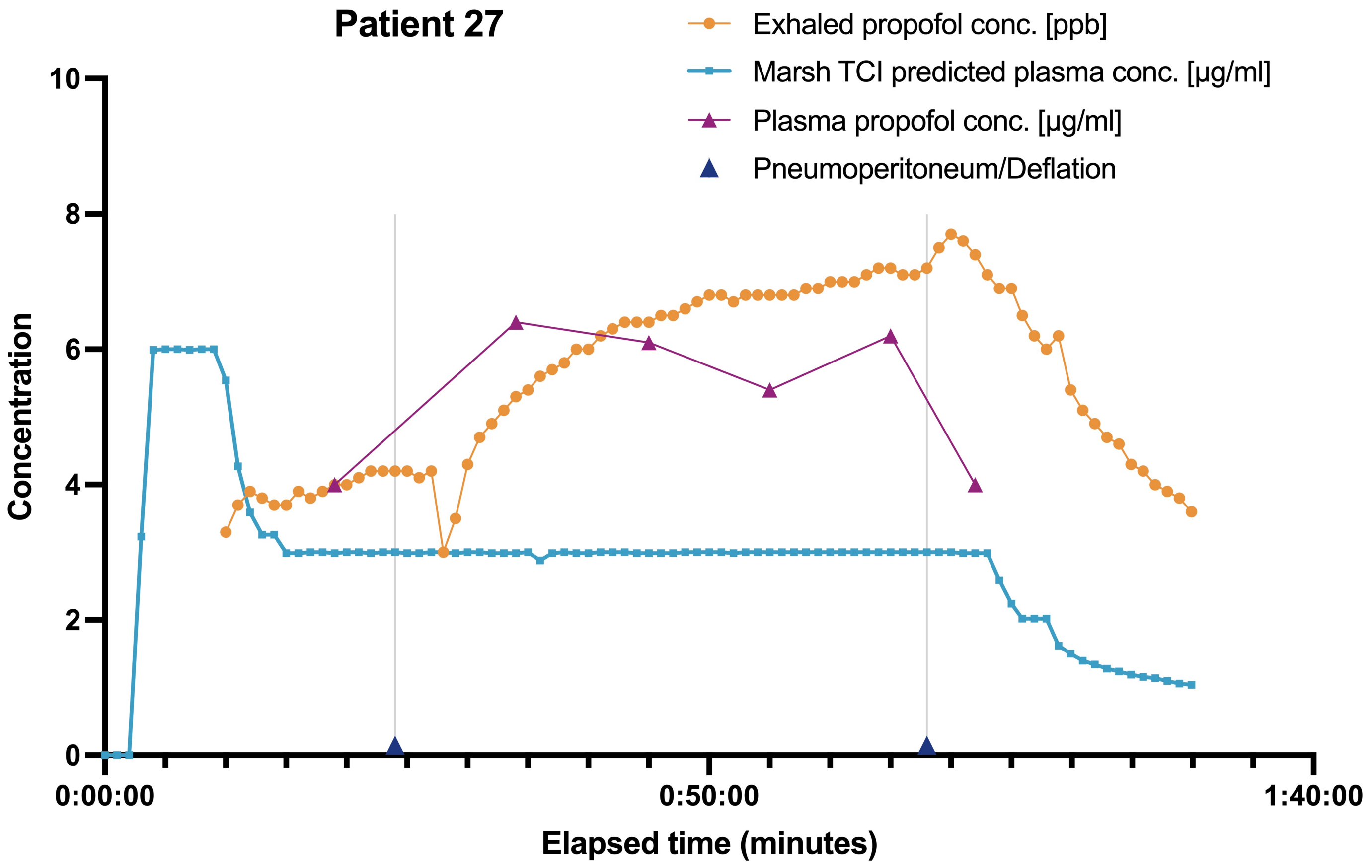
Patient 27
Exhaled propofol conc. [ppb]
Marsh TCI predicted plasma conc. [µg/ml]
Plasma propofol conc. [µg/ml]
Pneumoperitoneum/Deflation
Concentration
10
8
6
4
2
0
0:00:00
0:50:00
1:40:00
Elapsed time (minutes)

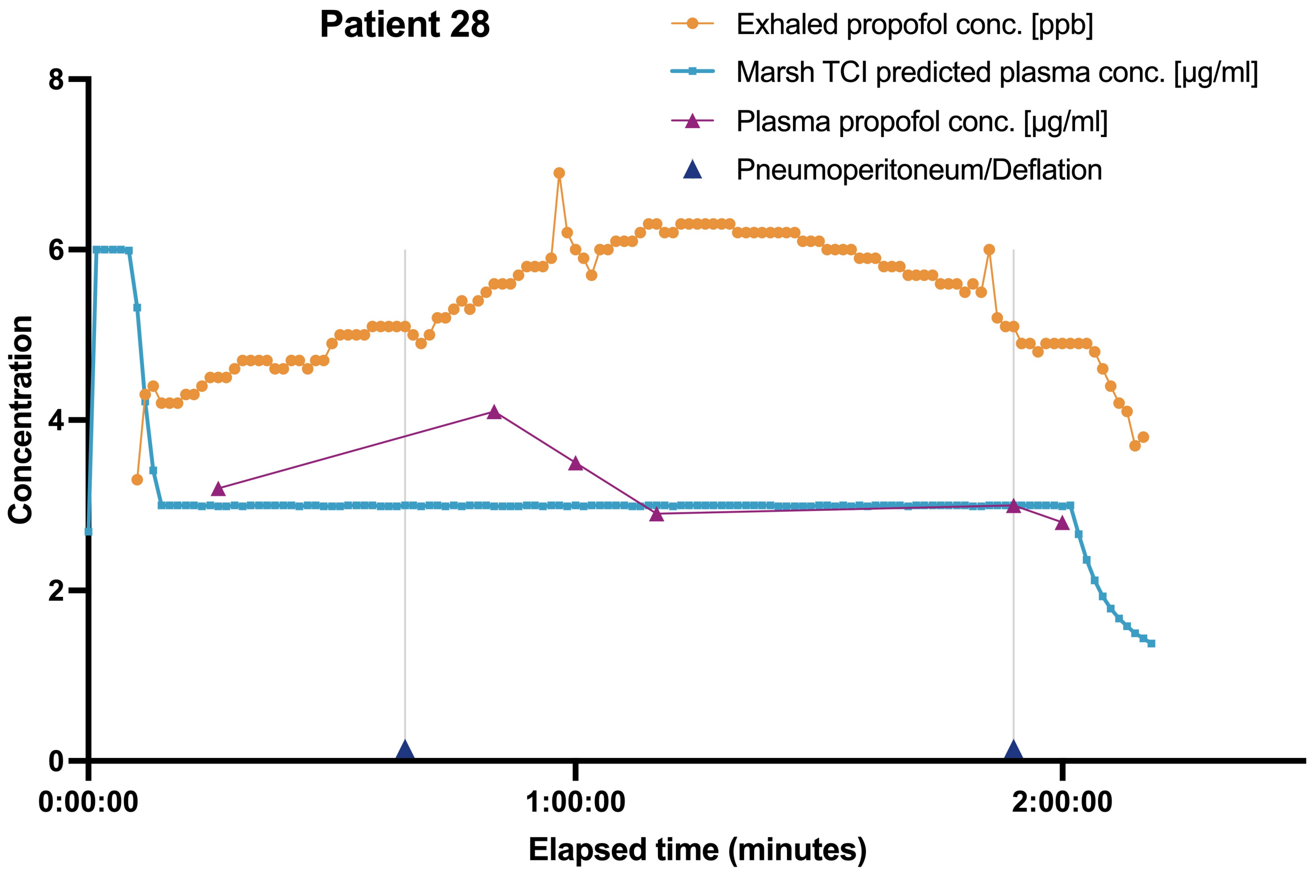

Patient 28
Exhaled propofol conc. [ppb]
Marsh TCI predicted plasma conc. [µg/ml]
Plasma propofol conc. [µg/ml]
Pneumoperitoneum/Deflation
Concentration
8
6
4
2
0
0:00:00
1:00:00
2:00:00
Elapsed time (minutes)

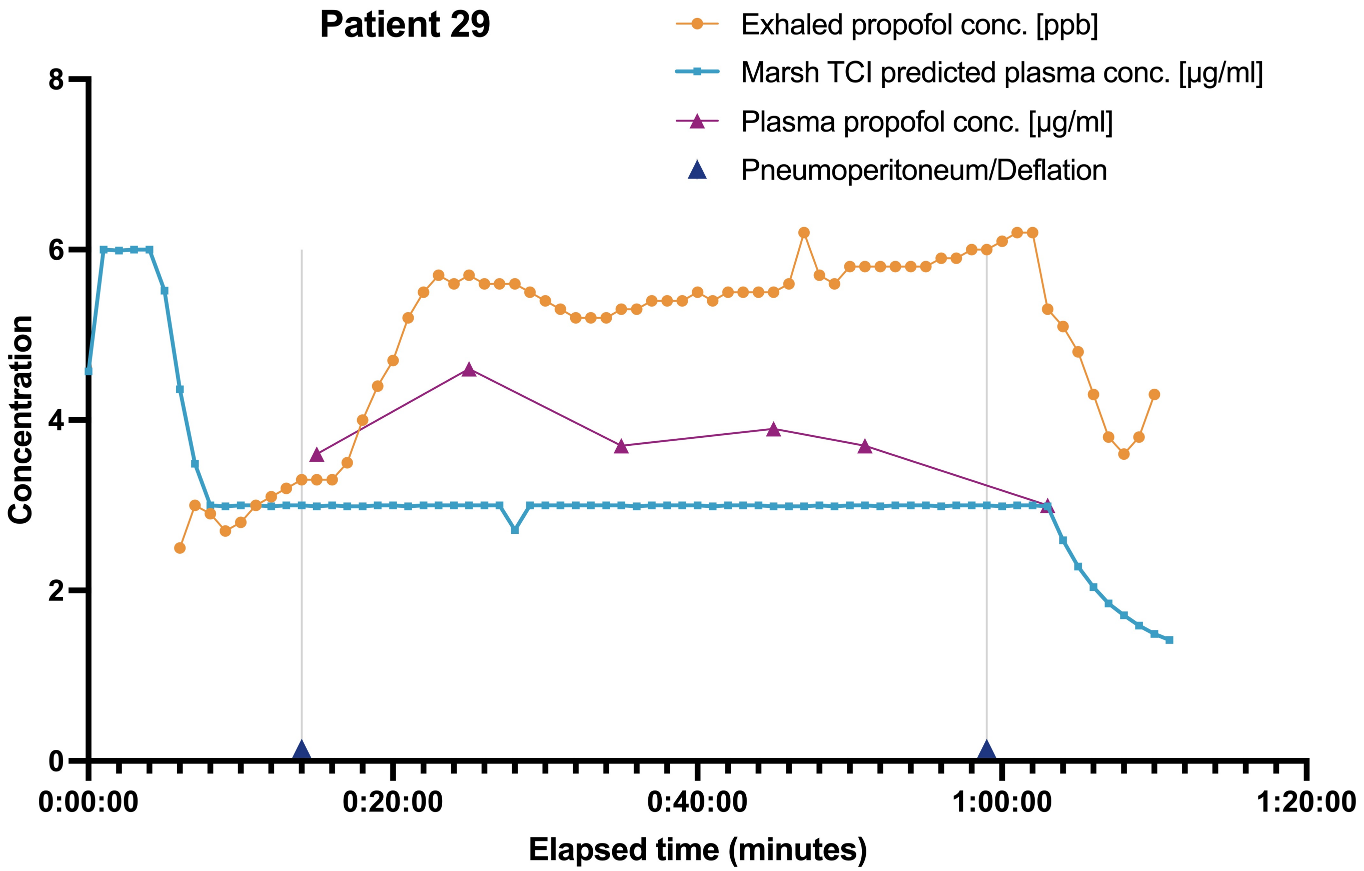

Patient 29
Exhaled propofol conc. [ppb]
Marsh TCI predicted plasma conc. [µg/ml]
Plasma propofol conc. [µg/ml]
Pneumoperitoneum/Deflation
Concentration
8
6
4
2
0
0:00:00
0:20:00
0:40:00
1:00:00
1:20:00
Elapsed time (minutes)
